# Touch may reduce cognitive load during assisted typing by individuals with developmental disabilities

**DOI:** 10.3389/fnint.2023.1181025

**Published:** 2023-08-03

**Authors:** Giovanni Nicoli, Giulia Pavon, Andrew Grayson, Anne Emerson, Suvobrata Mitra

**Affiliations:** ^1^School of Social Sciences, Nottingham Trent University, Nottingham, United Kingdom; ^2^Faculty of Social Sciences, University of Nottingham, Nottingham, United Kingdom

**Keywords:** cognitive load, developmental disabilities, autism, touch, light touch, executive function, cognitive-motor interference, assisted typing

## Abstract

Many techniques have attempted to provide physical support to ease the execution of a typing task by individuals with developmental disabilities (DD). These techniques have been controversial due to concerns that the support provider’s touch can influence the typed content. The most common interpretation of assisted typing as an ideomotor phenomenon has been qualified recently by studies showing that users with DD make identifiable contributions to the process. This paper suggests a neurophysiological pathway by which touch could lower the cognitive load of seated typing by people with DD. The required sensorimotor processes (stabilizing posture and planning and executing manual reaching movements) and cognitive operations (generating and transcribing linguistic material) place concurrent demands on cognitive resources, particularly executive function (EF). A range of developmental disabilities are characterized by deficits in sensorimotor and EF capacity. As light touch has been shown to facilitate postural coordination, it is proposed that a facilitator’s touch could assist the seated typist with sensorimotor and EF deficits by reducing their sensorimotor workload and thereby freeing up shared cognitive resources for the linguistic elements of the task. This is the first theoretical framework for understanding how a facilitator’s touch may assist individuals with DD to contribute linguistic content during touch-assisted typing.

## 1. Introduction

There have been several attempts to find strategies to physically support people with developmental disabilities (DD) in the generation of written linguistic content. The proposed methods include assisted typing (Lilienfeld et al., 2014; Jaswal et al., 2020), the rapid prompting method (Lilienfeld et al., 2014; Tostanoski et al., 2014) and facilitated communication (Wehrenfennig et al., 2008; Schlosser et al., 2014). Supported typing techniques usually involve an external assistant providing physical support at the level of the hand, arm, or shoulder of the user to help them point at objects or type on a keyboard.

Such interventions are adopted to enhance communicative options in people with various DD. They are generally introduced to people with little or no verbal communication skills, but the application of these interventions can persist even after the development of verbal communication. Although most general interest in the adoption of such techniques has focused on people with autism, assisted typing techniques have also been used in people with communication difficulties who have other DD, for example Down syndrome, Cerebral Palsy, and Fragile X.

These approaches are controversial (Mostert, 2001), however, as a range of studies have found that the user’s output is in most cases strongly influenced by the facilitator (Mostert, 2001, 2010; Wehrenfennig et al., 2008; Saloviita et al., 2014; Schlosser et al., 2014). Burgess et al. (1998) suggested that supported typing techniques were an example of the ideomotor effect (Carpenter, 1875) and Wegner et al. (2003) compared them to the episode of Clever Hans, the horse that reportedly answered mathematical questions with its hoof by reading subtle cues coming from his breeder. The ideomotor effect has since become the only widely discussed and accepted theoretical explanation of what occurs in supported typing (Schlosser et al., 2014; Saloviita, 2018). As a result, these techniques are currently not recommended as an assistive framework in formal education (NICE, 2012) although their practice has continued widely (Lilienfeld et al., 2014), and research on supported typing has persisted over the last two decades. Some authors have continued testing the authorship of written content through “message passing” tasks, showing that users largely fail to transmit information that the assistant does not have (Wehrenfennig et al., 2008; Saloviita et al., 2014). Others have used eye-tracking methods (Grayson et al., 2012; Jaswal et al., 2020) to show that users anticipatorily fixate the to-be-typed key, and so are actively involved in the process. Others have sought to assess and evaluate textual contents produced via such techniques adopting qualitative and quantitative linguistic analysis (Tuzzi, 2009; Saloviita, 2018), finding mixed results.

The results from the latter strand so far show that supported typing users have unique and idiosyncratic styles that differ from their assistants’, but style idiosyncrasy does not rule out the possibility of assistants influencing the content of the texts produced (Saloviita, 2018). Linguistic analysis conducted by Zanobini and Scopesi (2001), Emerson (2010), and more recently by Nicoli et al., (2021, under review), showed that textual output from supported typing contains linguistic patterns associated with both the assistant and the user. These results suggest that the whole process is best studied as a collaborative effort (Sartori and Betti, 2015; Curioni et al., 2019). Thus, considered as a possible conduit for a collaboration, supported typing methods present a more complex and nuanced picture than the apparent consensus discrediting them would suggest. In this perspective, it would be of interest to investigate whether there are neurophysiological pathways by which physically supporting people with DD such as autism (ASD) or Down syndrome (DS) could enable them to co-create textual output that they could not generate and express independently. It is possible that supporting the user by touch may reduce the cognitive and sensorimotor load of generating content and interacting with communication interfaces. Consideration of this possibility requires a thorough analysis of the computational challenges faced by individuals with a range of DD and whether or how supportive touch might ease them. The literature on supporting typing methods has not attempted this, and so doing this is a key purpose of this paper.

Typing is a complex skill that requires the coordination of multiple concurrent tasks. Typing while seated requires coordination of repeated reaching movements of the arm. The programming of these movements depends on postural control mechanisms that maintain a stable upright stance and counteract the mechanical perturbations produced by arm extensions (Section 2.1.1. “Mechanical base”). Postural control involves the integration of vestibular, somatosensory, and visual information, which in turn loads the executive function (EF) system (Section “2.1.3. Executive function load of sensorimotor coordination”). EF broadly refers to regulatory mechanisms that oversee goal-directed cognitive and motor processes by maintaining, and operating upon, task-relevant information (Baddeley, 1996; D’Esposito et al., 1999). Unifactorial models of EF attribute the roles of coordinating and regulating cognitive processes to a unique function, namely working memory or attentional control (Posner, 1980; Shallice and Burgess, 1991; Demetriou et al., 2019), whereas multifactor models distinguish between discrete, independent components (Diamond, 2013). Although the structure and unity of EF continue to be debated, it is generally agreed that EF is comprised of working memory, updating information held in working memory, mental flexibility (i.e., shifting between tasks or task sets), fluency, response inhibition, inhibition of task-irrelevant information, planning, problem-solving and self-monitoring (Miyake et al., 2000; Miyake and Friedman, 2012; Demetriou et al., 2019). See Section “6. EF deficits in DD” and Table 1 for EFs relevant to the seated typing task.

**TABLE 1:** Summary of the executive functions required during a typing task.

Executive function	Description
Inhibition	The ability to suppress irrelevant or distracting linguistic and non-linguistic information.
Updating	The capacity to continuously monitor and update information in working memory during the writing process. This involves integrating new ideas or information and modifying the written output accordingly.
Planning	The process of organizing and structuring the written content before typing. It includes developing an outline or framework for the text, deciding on the sequence of ideas, and formulating a plan for how to express those ideas effectively.
Self-monitoring	The ability to monitor one’s own performance during typing, checking for errors, inconsistencies, or deviations from the intended message. It involves self-evaluation and making adjustments to improve the quality and accuracy of the written output.
Working memory	The capacity to hold and manipulate information in mind while performing cognitive tasks. In typing, working memory is involved in temporarily storing and retrieving relevant information, such as graphemic strings, vocabulary, grammar rules, and previously written content.
Problem solving	The cognitive ability to identify, analyze, and solve problems encountered during the writing process. It includes identifying the most appropriate words, phrases, or sentence structures to convey meaning effectively.
Motor planning and coordination	The coordination of motor movements required for typing, including the precise control of finger movements and keystrokes to accurately produce written words.

The generation of the linguistic content to be typed places its own demands on EF (Section “2.2. Generating written content”) and planning the movement sequence required to type out that content also comes with its own EF load (Section “2.1.2. Motor planning” and Section “2.1.3. Executive function load of sensorimotor coordination”). The concurrent demands of sensorimotor coordination and cognitive tasks on common computing resources (resulting in interference in some conditions) has been extensively researched in the past few decades, particularly in the context of aging (Section “3. Cognitive-motor interaction”). The literature has also documented the beneficial impact an external somatosensory signal can have on postural control. The possibility we present here is that supportive touch might reduce the computational workload of generating and typing linguistic content by assisting the sensorimotor processes of typing (Section “4. The utility of touch information in balance control”). Given the noted interactions between such sensorimotor and concurrent cognitive processes, tactile support has the possibility of freeing up cognitive capacity for the required linguistic effort. This prospect is considered in the context of extensive evidence that a range of DD affect both motor coordination and cognitive capacity. As we expand in Sections “5. Sensorimotor deficits in DD” and “6. EF deficits in DD,” individuals with developmental disabilities (DD) experience sensorimotor deficits that affect both motor coordination and cognitive capacity. Sensorimotor deficits include impairments in sensory integration, postural control, and difficulties in planning and controlling voluntary movements. These deficits, combined with weakened trunk stability and impaired sensory integration, can make it challenging for individuals with DD to maintain a stable seated position and perform the precise arm movements required for typing. Furthermore, individuals with DD often experience executive function (EF) deficits, which may impact cognitive processes involved in typing, such as working memory, planning, flexibility, and inhibition. Thus, acknowledging the simultaneous presence of sensorimotor and EF deficits in DD populations sets the context for interpreting and implementing interventions such as light touch that be effective primarily by reducing the workload associated with the sensorimotor component.

In Section “2. Task analysis,” we start providing a detailed analysis of what typing while sitting requires from a sensorimotor and cognitive perspective. Here, we also show how the associated sensorimotor and linguistic actions significantly draw on EFs. In Section “3. Cognitive-motor interaction,” we present the literature on cognitive-motor interaction (CMI) that shows how concurrent motor and cognitive tasks can interfere due to shared cognitive resources. In Section “4. The utility of touch information in balance control,” we present the literature on the role that touch can play in aiding postural control, thereby reducing cognitive load. In Section “5. Sensorimotor deficits in DD,” we establish the relationship between DD and sensorimotor deficits, and then, in Section “6. EF deficits in DD,” we demonstrate how a range of DD limit EF capacity. In Section “7. General discussion−Sequences of events,” we synthesize the assembled information in support of the proposal that touch-based physical support may facilitate typed communication by individuals with DD by reducing the computational load of the sensorimotor and cognitive components of the task.

## 2. Task analysis

As typing is a complex task that involves both sensorimotor and cognitive components, in this section we analyze each component in turn, with a particular focus on how executing and coordinating these bear on the cognitive workload. We start with the sensorimotor coordination required for pointing gestures of the arm, and consider the cognitive processes involved in generating the linguistic content to be written.

### 2.1. Sensorimotor coordination

The task of typing on a keyboard involves repeatedly aiming the typing fingertip at the required key and making a reaching movement toward the key. We assume that the key readily provides haptic and visual feedback when it is pressed. These sensory signals indicate goal achievement, and therefore the termination of the key press action.

Leaving aside the linguistic aspect, the action of typing requires the coordination of a postural task (setting the mechanical base) and a manual reaching task (pointing at the keyboard). The postural task stabilizes the torso, and the torso’s position and velocity affect the programming of the arm’s reaching trajectory (Thelen and Spencer, 1998). To better understand the latter task’s dependence on the former, we must examine the information requirements of both task components.

#### 2.1.1. Mechanical base

The informational support for maintaining the body’s upright stance comes from the integration of perceptual data processed by vestibular, somatosensory (cutaneous, proprioceptive, and joint receptors) and visual systems (Allum and Keshner, 1984; Diener et al., 1988; Shumway-Cook and Woollacott, 2012). The vestibular system provides information about the head’s movement with respect to gravity and inertial forces (Horak et al., 1994). The somatosensory system provides information about the body’s position and motion with respect to support surfaces and about the dynamic inter-relationship between body segments (Diener et al., 1984; Roll and Roll, 1988). The role of vision is to provide information about the position and motion of the head relative to environmental objects and a sense of verticality. Visual information is particularly salient in the detection of self-motion as movements of the head through a visible environment generate flows of optical elements across the entire visual field (Lee and Lishman, 1975; Dijkstra et al., 1992). Anterior-posterior head motion produces radial optical flow whereas medial-lateral head motion generates lamellar flow (Warren, 2010). When these visual signals are available, the body sways less in both planes (Edwards, 1946; Paulus et al., 1984, 1989). Indeed, the ratio of body sway between eyes-open and eyes-closed conditions, the Romberg quotient, is a clinical indicator of postural stability (Romberg, 1853). Research suggests that the maintenance of balance (for example, keeping the body’s center of mass within the base of support) involves both exploratory and corrective body sway. Exploratory sway generates perceptual information (including optical flow) that guides the compensatory sway that corrects drifts toward instability (Riccio et al., 1993; Riley et al., 1997). Although visual information is not essential for maintaining balance, it plays a dominant role when it is available and provides a strong signal of head motion. For example, when the train in the next track moves off, standing passengers in a static train produce a postural reaction consistent with their own train setting off. This happens despite the absence of any self-motion signals from their vestibular and somatosensory systems. In upright position, body sway is lower in the presence of vision, a result commonly interpreted as indicating greater stability (Andersen and Dyre, 1989; Masson et al., 1995). The closer the environmental objects on which the eyes fixate, the less the body sways (Lee and Lishman, 1975; Dijkstra et al., 1992). As the optical flow produced by nearer objects is larger in magnitude, this suggests that optical flow information is actively used to maintain stance stability.

In the typing task context, the posture control system has a dual function: a stabilizing function in maintaining upright stance in the seated position, and a task-facilitative (Stoffregen et al., 2000) function that: (a) controls the position of the mechanical base (shoulder) from which the arm extension is parameterised, (b) contributes to the reaching action by controlling forward lean, and (c) anticipates and adapts to the perturbation generated by arm extension. The direction and amplitude of the reaching gesture appear to be prepared before movement begins, and, for this to be possible, information about the initial position of the limb is important (Polit and Bizzi, 1979). The position of the shoulder at the start of each reaching trajectory is the result of the facilitatory and stabilizing functions of the posture control system. Both these functions are confirmed (in work on standing balance) by the activation of leg muscles during anticipatory postural adjustments before arm movement (Shepherd et al., 1993), and by the occurrence of earlier and larger postural adjustments reaching distance increases and the support area shrinks (Moore et al., 1992). With regard to planning the direction of pointing movements, there is considerable evidence that the trajectories are planned in spatial coordinates (Tresilian, 2012; Bosco et al., 2017). How this operates is addressed in the next section, but here it is important to note that the development of an internal coordinate system, be it head-, hand- or body-centered, requires a reliable and stable origin for reference. The observer’s posture can influence the spatial relationships between objects in their visual field and can also affect their ability to perceive depth and movement. Additionally, changes in posture can alter the visual cues that are used to perceive the environment, such as the relative size and position of objects. Body sway, which refers to the small movements of the body caused by changes in balance and weight distribution, can have an impact on the visual coordinate system by altering the relative positions and orientations of the observer and the objects in their visual field (Thelen and Spencer, 1998).

This section discussed the sensorimotor functions of maintaining an upright stance that is stable and able to support the motor functions of the task at hand. The next section outlines the components of arm trajectory planning in a task like typing.

#### 2.1.2. Motor planning

The first element of planning the trajectory of the fingertip (the working point) to the required key is visually locating the target key (termed “visual regard”). This involves coordination of the trunk, head, and eyes (note that all three are also involved simultaneously in postural control). Forming the trajectory involves a differencing mechanism that compares the working point’s current position to its required position. This internal feedback signal (internal to the trajectory generator) is used integratively to drive the current position of the working point to its target location (Saltzman and Kelso, 1987; Bullock et al., 1993; Hoff and Arbib, 1993). This process is one of negative feedback control and requires at least intermittent sampling of visual and proprioceptive information about the working point’s current and required position in space. A differencing mechanism like this can only operate accurately if both the current and required positions are represented in the same coordinate system. These locations in space could be monitored in a coordinate system located at the visual egocenter (Tresilian, 2012), but it has been suggested that the coordinate system is centered on the point in the visual field where the eyes are fixating (Shadmehr and Wise, 2005). As this makes eye fixation fundamentally important to trajectory planning, we recall that it also affects the sampling of optic flow from eye-head motion and proprioceptive information from ocular vergence for use in postural control.

Aside from feedback-based closed loop control, fast movements also exhibit open loop control in which the trajectory is specified without feedback at execution time (Adams, 1971). Both these types of control have also been suggested for postural control (Collins and De Luca, 1993). Most reaching movements exhibit a hybrid control pattern whereby open loop control takes the working point close to the target location. Then, a set of sub-movements controlled in closed loop brings the working point precisely to the target location (Meyer et al., 1988). Accurate open-loop control is only possible if the consequences of motor commands can be estimated in advance. This implies access to detailed information about the articulatory apparatus. This process operates in a feedforward manner, utilizing predictions of the results of motor commands and preparing elements of the controlled system for the resulting changes in their states (Pisotta and Molinari, 2014). The need for system knowledge also emerges when considering the transformation of spatial trajectory information to the required angular motion of the body’s joints (inverse kinematics) and the muscle activity required to achieve these. Predictions of the sensory consequences of planned motor commands are termed forward models (Miall and Wolpert, 1996).

#### 2.1.3. Executive function load of sensorimotor coordination

Research agrees that there is a cognitive workload associated with balance, gait, or other goal-directed sensorimotor coordination. In many situations, performing these tasks concurrently with other cognitive tasks can pose significant challenges. This may be due to capacity-limited cognitive resources being shared by both types of tasks (Mitra, 2004) or because tasks are functionally linked in terms of performance or system requirements (Stoffregen et al., 2007). Maintaining the body’s balance is the key imperative for postural control, but posture and gait are constantly modulated in service of supra-postural tasks. The latter function of the posture control system is likely more demanding of cognitive resources than simply retaining balance as it must link and adapt coordination to specific and transient task goals.

As seen in the previous section, keeping an upright stance is the result of sensory and motor processes. Sensory processes compute the position of the body in space, motor processes allow for muscle and movement adaptation and responses to the detected position. The sensory processes rely on the integration of visual, vestibular and proprioceptive sensory information. The information provided by each of these channels is then weighted to give precedence according to reliability (Shumway-Cook and Horak, 1986). The literature clearly shows that the integration of sensory information poses demands on cognitive resources, so that decreased postural control, due to injury, aging or sensorimotor deficits (Fournier et al., 2014) increases the demands for attentional resources to maintain the body’s balance. When information provided by one of the sensory channels deteriorates, the demands for attentional resources to deal with postural tasks increases (Shumway-Cook and Woollacott, 2000; Mahboobin et al., 2007). A striking implication of these studies is that adding reliable sensory information may substantially decrease the demands for executive resources that can then be allocated elsewhere. Thus, given the essential role of posture control in the execution of supra-postural tasks, by extension, multisensory integration also plays a decisive role in the planning and execution of movements (Betti et al., 2021). Motor planning itself draws on executive and cognitive resources. It has been proposed that motor planning and executive functions are two distinct heterogeneous domains of cognitive functioning (Wunsch et al., 2016), but many studies have reported dual-task interactions between motor planning and (visuo-spatial) working memory with detrimental effect on the latter (Schütz and Schack, 2020). Deficits in motor planning and execution, reported in many DD (Mari et al., 2003; Cummins et al., 2015; Alesi and Battaglia, 2019; Studenka and Myers, 2020), would therefore pose increased demands on the executive/cognitive domain.

The internal models involved in motor task execution are the suggested means of several important functions in motor control. These include motor learning, separating the effects of self-motion from sensory input, and counteracting the impact of delays in neural signal transmission (Miall and Wolpert, 1996; Wolpert and Kawato, 1998). In terms of neural mechanisms, the planning and execution of reaching arm movements involves the parietal and premotor areas in spatial planning, the primary motor cortex and descending pathways in the activation of muscle groups, and the basal ganglia and cerebellum in refining the process by accounting for the current and predicted states of the body (Shumway-Cook and Woollacott, 2012). The cerebellum is thought to be involved in the use of both inverse models (providing the neural commands required for a given trajectory) and forward models predicting sensory consequences of actions (Wolpert et al., 1998). Wolpert and Kawato (1998) suggest that multiple pairs of forward and inverse models need to be trained during the acquisition of motor skills such that models suited to given task conditions can be activated, or control can be switched to better suited models if conditions change. Note that both selection and switching are EFs (Miyake et al., 2000).

As the reaching arm’s position is critical in trajectory planning and depends on both the stabilizing and task-facilitative roles of postural control, postural states and actions (e.g., of the torso) must be included in internal models of the kind of seated typing movements we are considering here (Wolpert and Kawato, 1998; Morasso et al., 1999; Kuo, 2005). A key point with respect to both limb movement and postural control is that much of it is anticipatory in nature. Whether responding to an expected external perturbation, or supporting a voluntary movement that perturbs balance, the required postural adjustments must be estimated and applied in advance. Evidence of this is seen not only in the case of executed movements (Belen’kiĭ et al., 1967; Bouisset and Zattara, 1988), but also in the case of motor imagery where only movement planning occurs but not execution (Wider et al., 2020, 2022). The close coordination that has been observed between anticipatory postural adjustments and associated limb movements (e.g., these adjustments can be affected independently by the magnitude of perturbation and magnitude of the action triggering the perturbation) has led to suggestions that anticipatory postural adjustments must be integral elements of limb movement planning (Aruin and Latash, 1995, 1996). As the limb movements themselves also involve anticipatory components within internal models, and anticipatory processes must involve choices based on task conditions and memory, the central importance of EFs in the selection, planning and execution of the seated typing movements we have been considering becomes evident.

This section established that sensorimotor coordination of both postural control and focal movement planning components of seated typing make significant demands on cognitive resources such as EFs. The next section considers the simultaneous EF demands of the content generation aspect of the seated typing task. Both these discussions should be taken in the context of EF deficits in DD that will be outlined in Section “6. EF deficits in DD.”

### 2.2. Generating written content

Writing is a problem-solving task (Cornoldi et al., 2010) that requires the coordination of multiple operational procedures working at central and peripheral levels (Ellis, 1982). In this section, we broadly describe the main processes involved in the generation of a written output with a particular focus on the demands these processes load on EF capacity.

#### 2.2.1. Writing operations

The operational procedures involved in a writing task can be broadly divided into three recursive phases (Hayes and Flower, 1980). Models can vary in the number, name, and attribute of these phases. For present purposes, we present a broad summary. Note that while these processes are hierarchically ordered in a cascade-like model, their execution is intertwined in time (McCutchen, 2000; Cornoldi et al., 2010; Purcell et al., 2011). Thus, writing being a much slower task than talking, the processes that happen higher in the route (the generation of contents) may be expanded, modified, or updated during the completion of the lower (content’s transcription). The first operational phase is the retrieval of semantic or contextual information and formulation of the ideas that need to be organized in text form (Hayes and Flower, 1980; Ellis, 1982; McCutchen, 2000; Cornoldi et al., 2010). This phase is constantly updated during the other phases of writing although an outline plan is needed in very first stages to allow the writing process to start (Cornoldi et al., 2010). The second phase translates into linguistic representations of the ideas generated in the first phase (Berninger, 1999; McCutchen, 2000; De Vita et al., 2021). This process can be itself split into two components: a text generation phase, where concepts are translated into lexical units and the broad text is organized into a syntactic plan, and a transcription phase where these linguistic representations are transformed into written words (Cornoldi et al., 2010; De Vita et al., 2021). The latter is composed by a central and a peripheral process. The central (spelling) process creates a graphemic and orthographic representation of the linguistic representation. The peripheral (motor) process realizes graphemes through handwriting (graphomotor skills), typing (pointing gestures) or oral spelling (Ellis, 1982; De Vita et al., 2021). The third phase involves revising procedures, namely operations that check textual adequacy and linguistic features (Hayes and Flower, 1980; Berninger, 1999; Cornoldi et al., 2010).

#### 2.2.2. Executive function load of writing

A writing task requires constant and recursive shifting between long-term memory retrieval (knowledge) and textual operations (processes), (Hayes and Flower, 1980; Cornoldi et al., 2010). The coordination of this multiple, hierarchically ordered, and intertwined operations are overseen and constrained by an executive system (Berninger, 1999; McCutchen, 2000). We have already referred to writing as a problem-solving task (problem solving is an EF, see section “6. EF deficits in DD” for references), but inhibition, updating, planning, self-monitoring and working memory are also involved (Salas and Silvente, 2020; De Vita et al., 2021). Please refer to Table 1 for a summary of the EF involved in a writing task. Depending on the level of expertise, the constraints of EF are dealt with differently. Expert writers utilize much of their EF resources on idea generation, conceptual organization, and retrieval of lexical and syntactic structures suited to the context and the goal of the task. They are also able to coordinate the revision process to both check orthographic, linguistic, and overall general textual aspect (Cornoldi et al., 2010). Less expert writers such as children devote much of their working memory resources to spelling and motor processes, as these operations are not yet automatised, resulting in less resources being available for semantic and linguistic planning (Berninger, 1999; McCutchen, 2000; Salas and Silvente, 2020; De Vita et al., 2021) and greater challenges in coordinating the revision of orthographic and linguistic features (Cornoldi et al., 2010). What clearly emerges from the literature on writing processes is that to perform successfully at a good level, cognitive resources have to be devoted to higher level operations (text generation, concept planning, writing oversee and monitoring), rather than on lower ones (orthographic planning, motor execution). Struggling to derive an orthographic buffer from linguistic representations or else to coordinate translation into motor gestures of the output of the orthographic buffer would overload the working memory system with a detrimental effect on higher-ordered operations (Berninger, 1999; Cornoldi et al., 2010; Salas and Silvente, 2020; De Vita et al., 2021).

## 3. Cognitive-motor interaction

Section “2. Task analysis” provided an analysis of the computational components of the task of seated typing. The load placed on EF by both the motor and linguistic production components was noted. This section summarizes the extensive literature on interactions between motor coordination and concurrent cognitive tasks. The essential point it seeks to establish is that EF capacity can be a limiting factor in the success of cognitive-motor dual tasks. Seated typing is, of course, a clear instance of this type of dual task.

It is increasingly clear that sensorimotor coordination involved in posture and gait control, not to mention the planning and execution of voluntary goal-directed movement, draws significantly and continuously on EF resources (Fraizer and Mitra, 2008b; Al-Yahya et al., 2011; Amboni et al., 2013). This understanding arose initially in research on the effects of aging on balance and gait control. Aging is associated with both reduced EF capacity (Fisk and Sharp, 2004; Clarys et al., 2009) and increased involvement of higher-level cognitive processes in motor coordination (for example, increased reliance on visual feedback) (Lajoie et al., 1996; Peper et al., 2012; Yeh et al., 2014; Hollands et al., 2017). A large body of dual-tasking research has shown that adding attention and EF load to ongoing balancing or gait tasks affects performance in either or both tasks (Li and Lindenberger, 2002; Woollacott and Shumway-Cook, 2002; Fraizer and Mitra, 2008a; Al-Yahya et al., 2011). Such dual-task interactions have been found not only in older people and neurological patients, but also in healthy young adults. This suggests that these cognitive-motor interaction effects do not arise from the EF capacity and sensorimotor performance deficits associated with old age (or neurological conditions) but are largely amplified by them.

Several theoretical frameworks have been used to explain the complex results obtained across studies of cognitive-motor interaction. The most commonly adopted framework accepts that there is some involvement of high-level cognitive function in posture and gait control (Tresilian, 2012), and cognitive-motor dual-task interactions arise because both types of tasks engage common mechanisms. Drawing on classic attention theory (Broadbent, 1958), the bottleneck version of this account suggests that cognitive-motor interaction results from the sharing of a serial processor between cognitive and motor operations (Pashler, 1994; Meyer and Kieras, 1997; Tombu and Jolicoeur, 2003; Bayot et al., 2018). Operations that utilize the same neural pathway or network must take turns. The bottleneck account has two variants, structural and strategic (Bayot et al., 2018). The former suggests that the processor generates a bottleneck effect at the decision-making stage, whereas the latter postulates that the same interference happens at the response-control stage or at a peripheral level when tasks share the same input or output processors. The capacity or resource sharing model (Tombu and Jolicoeur, 2003; Mitra, 2004; Bayot et al., 2018) posits that a finite pool of processing resources must be shared by concurrent task operations. If the resource draw of one task increases, a deficit in resourcing the other emerges. Accordingly, older people in particular have been shown to operate a “posture first” principle (Shumway-Cook et al., 1997) whereby they prioritize the balancing task by discontinuing a concurrent cognitive task when they detect a risk of balance failure. Some accounts postulate multiple resource pools specific to particular types of operation (e.g., spatial processing) such that interactions occur when cognitive and balancing tasks place demands on the same type of processing resource (Navon and Gopher, 1979; Bayot et al., 2018).

In cognitive-motor interaction research, motor performance is commonly evaluated using speed, cadence, or stride time to assess gait, or center-of-pressure (the point of application of ground reaction force) displacement and frequency to measure body sway to assess postural control (Fraizer and Mitra, 2008a; Al-Yahya et al., 2011). Slower gait speed, reduced cadence, or increased body sway are taken to indicate a deterioration in motor performance. A wide range of cognitive tasks including working memory, verbal fluency, inhibition, set-shifting and arithmetic skills, most involving EF, have been studied using behavioral performance indicators such as response time and accuracy, and, more recently, at the neurophysiological level using electrophysiology (Swerdloff and Hargrove, 2020; Reiser et al., 2021).

Despite variability in outcomes, sometimes due to methodological differences (Fraizer and Mitra, 2008a; Bayot et al., 2018), research has tended to show that concurrent EF tasks result in performance deficits in balance and gait in older people (Shumway-Cook et al., 1997; Morris et al., 2000; Swanenburg et al., 2009; Chang et al., 2010; Nadkarni et al., 2010; Al-Yahya et al., 2011; Patel et al., 2014; Fernandes et al., 2015; Bahureksa et al., 2017; Hsiu-Chen et al., 2020; Morenilla et al., 2020; Przysucha et al., 2020; Varas-Diaz et al., 2020). Similar results have been reported in stroke patients (Lee et al., 2020) and, importantly, also in young adults (Dault et al., 2001; Woollacott and Shumway-Cook, 2002; Pellecchia, 2003; Nadkarni et al., 2010; Onofrei et al., 2020) and children (Chang et al., 2010; Bucci et al., 2013; Palluel et al., 2019). The literature also describes executive task performance deficits when body posture is perturbed or motor task complexity increases (Andersson et al., 1998, 2002; Brown et al., 1999; Yardley et al., 2001; Wollesen et al., 2016; Estevan et al., 2018; Abou Khalil et al., 2020; Stephenson et al., 2020; Swerdloff and Hargrove, 2020; Reiser et al., 2021). These results appear to provide clear evidence that the coordination of balance or gait has an EF load associated with it. A reduction in available EF resources negatively affects coordination, and impaired coordination adds to EF load.

Although the results of cognitive-motor dual tasking are most often interpreted as patterns of mutual interference, in some situations, there are reasons to be wary about such conclusions (and the theories they are taken to support). When adding a concurrent sensorimotor task, the accuracy of a cognitive task is reduced, or the response time increases, it would appear clearly that dual tasking negatively impacted cognitive task performance. Where the sensorimotor task is simply maintaining upright stance, the effect of a concurrent task is measured as a change in body sway. Commonly, increased sway is interpreted as a negative effect on postural stabilization functions. By this logic, reduced body sway would indicate improved stability. Although commonly applied, this logic does not adequately explain all empirical data. Posture-cognition dual task studies have reported both increased and reduced body sway when performing concurrent cognitive tasks (Fraizer and Mitra, 2008a). It is also doubtful that the posture control system always cares to reduce body sway to increase stance stability beyond simply ensuring that the center of gravity stays within the base of support (Stoffregen et al., 1999). If so, a change in body sway may indicate an imperative other than improving stance stability. The logic also fails where the secondary task engages the posture control system in facilitative actions other than, and even in opposition to, maximizing stance stability (Stoffregen et al., 1999). If the secondary task requires precise eye fixations for reading, for example, body sway might be reduced, not because reading impedes the posture control system’s stability maintenance function, but because postural control acts to stabilize the head to aid reading (Stoffregen et al., 2000). Such considerations underpin an alternative view of posture-cognition dual tasking that points to functional linkages (such as the shared use of vision) rather than a competition for cognitive resources as the mechanism of interaction (Stoffregen et al., 2007). This approach also emphasizes the fact that postural control in everyday life is almost always organized to enable some supra-postural tasks rather than simply to maximize stance stability. Thus, postural control is itself a multi-task function charged with maintaining stance as well as facilitating supra-postural tasks.

Many of the ambiguities about the effects of cognitive load on motor tasks arise in laboratory studies of dual tasking in which the postural task is simply to maintain upright stance. In some situations, the cognitive task clearly uses a function that postural control also uses, for example, when the cognitive task requires visual perception and attention that are also required by a balancing task. But in other cases, the cognitive task has no obvious sensorimotor component. Interaction effects have been reported in both cases, but their consistency and size under the latter conditions have been questioned (Stoffregen et al., 2007). Using the results of dual task studies to draw conclusions about the role of cognition in the control of quiet standing may not be straightforward, but the effect of concurrent cognitive load is more clearly detrimental when counteracting perturbations to body posture (Fraizer and Mitra, 2008a). There is considerable evidence of cortical involvement in shaping responses to postural perturbation, including the modulation of postural response based on cognitive state, sensorimotor conditions and past experience (Jacobs and Horak, 2007; Jacobs, 2014; Bolton, 2015; Peterson et al., 2016; Ghosn et al., 2020). Switching attention (an EF) to balancing function is a key aspect of responding to perturbation (Maki et al., 2001). Concurrently performing EF tasks (e.g., mental arithmetic) reduces the amplitude of postural muscle activity (though not its latency) when responding to perturbation (Rankin et al., 2000). Analogously, when attention is engaged in a tracking task during postural perturbation, the magnitude (but not the latency) of the cortical response, detected electrophysiologically as the N1 potential (Adkin et al., 2008), is attenuated (Quant et al., 2004). Deterioration in EF predicts loss of balance in older individuals (Buracchio et al., 2011; Kearney et al., 2013) and concurrent performance of EF tasks affects a number of gait measures (Al-Yahya et al., 2011). In the case of gait, the prefrontal cortex is known to become involved when the coordination needs to adapt to changing task requirements (e.g., a change of speed or a transition to running) (Suzuki et al., 2004), suggesting a role for EF in tailoring coordination to task goals.

The effects of concurrent cognitive load become even clearer when the motor task includes aimed movements of the upper limb as everyday tasks like driving (Strayer and Johnston, 2001; Recarte and Nunes, 2003). Pursuit-tracking tasks have a long history of use in dual-task interactions (Isreal et al., 1980; Kramer et al., 1983; Brown, 1998; Gazes et al., 2010), including as a simulated driving task (Strayer and Johnston, 2001) and as a secondary task during postural perturbations (Mcilroy et al., 1999; Norrie et al., 2002). Baker et al. (2018) monitored the electrophysiological correlates of detecting and tallying the occurrence of visual oddball stimuli while performing a visuomanual tracking task. They found that adding the tracking task attenuated the markers of attentional (but not perceptual) processes even though tracking task performance was itself unaffected by the oddball task at these timescales. Tracking performance did suffer when, after detecting an oddball, the target tally was updated (updating is recognized as an EF). This demonstrates that cognitive-motor dual task interactions can have intricate mechanisms composed of separate, asymmetric and asynchronous influences between tasks.

For present purposes, the key message from this research is that there is a cognitive workload associated with balance, gait, or other goal-directed sensorimotor coordination. In many situations, performing these tasks concurrently with other cognitive tasks can pose significant challenges. This may be due to capacity-limited EF resources being shared by both types of tasks, or because task pairs are functionally linked in terms of performance requirements. Maintaining the body’s balance is the key imperative for postural control, but posture and gait are constantly modulated in service of supra-postural tasks. The latter function of the posture control system is likely more demanding of cognitive resources than simply retaining balance as it must link and adapt coordination to specific and transient task goals. These considerations support our present focus on the potential benefits of facilitating motor coordination as a means of easing the combined workload faced by individuals with DD when they try to communicate by typing text.

## 4. The utility of touch information in balance control

A number of techniques seek to assist individuals with DD to type text on a keyboard or to point to textual or pictorial information on a screen by providing them with supportive touch on the torso or arm (Lilienfeld et al., 2014; Schlosser et al., 2014; Beals, 2022). Critics of these systems have pointed out that touch information can serve to cue the typing actions (Wegner et al., 2003; Mostert, 2010; Saloviita, 2018) but the literature on supported typing has not considered the possibility that, different from specific action-cuing, an external touch signal can aid postural control and thereby reduce the overall computational workload of the typing task.

The role of touch in balancing has been investigated in detail in the context of balance challenges due to aging (Jeka, 1997; Johannsen et al., 2009; Rabin et al., 2013; Ditthaphongphakdee and Gaogasigam, 2020), stroke (Lee et al., 2018, 2020; Martinelli et al., 2018), blindness (Jeka et al., 1996; Schieppati et al., 2014) and childhood DD (Baldan et al., 2014; Chen and Tsai, 2015; Chen et al., 2019). The benefits of light touch in balancing have also been demonstrated in healthy young adults (Krishnamoorthy et al., 2002; Magalhães and Kohn, 2011; Martinelli et al., 2018; Kaulmann et al., 2020), but its impact becomes greater in the context of motor and postural difficulties due to disability or aging (Baldan et al., 2014). Early research on this showed that light touch (< 1N of force applied) can improve postural stability (as indicated by reduced body sway) and reduce falling risk (Holden et al., 1994; Jeka and Lackner, 1994, 1995; Jeka, 1997). Light touch does not support the body’s weight but aids postural control by providing an external somatosensory reference for judging body motion (Jeka and Lackner, 1995; Riley et al., 1997). The touched object need not even remain static against the applied force. Lightly touching a hanging curtain can replace the level of reduction in body sway that the availability of vision provides (Riley et al., 1999). Indeed, light touch can be as effective as forceful touch in stabilizing body sway by providing information to guide anticipatory muscle activation (Jeka and Lackner, 1994). Importantly for present purposes, research shows that postural assistance by touch works whether the touching is active (for example, the assisted person touches the external surface) or passive (for example, another individual or a mechanical arm lightly touches the individual on the back or shoulder) (Johannsen et al., 2009, 2011).

Light touch can also reduce postural instability arising from to a concurrent cognitive task (Lee et al., 2018, 2020) suggesting that light touch may reduce the overall cognitive workload of cognitive-motor dual tasking. Chen et al. (2015) showed, for example, that light touch to aid posture control resulted in improved performance in a concurrent visual search task. Such results suggest that light touch may play a particularly important role in reducing visual attention load during cognitive-motor dual tasking. In this context, it is important to note that touch information can be used by the posture control system to facilitate a supra-postural task such as visual search, as in the case of Chen et al. (2015), and maintenance of the light touch itself, as in the case of Riley et al. (1999). The latter study found reduced postural sway only when precisely touching a hanging curtain was an actual task goal. For present purposes, the point is that touch information can reduce the total workload of a seated typing task, potentially by assisting the maintenance of upright stance, or by facilitating the postural component of planning and executing the repeated reaching movements of the typing arm. To the extent that these coordination tasks involve EF, reducing their workload has the potential to release resources for the message formulation and sequencing functions involved in typed communication. Section “4. The utility of touch information in balance control” presented the details of the visual system’s dual role in the maintenance of postural stability and in postural facilitation of other tasks. This background now enables us to appreciate, in the context of assisted typing, the potentially key role of a touch signal in reducing the demands placed on the visual system by postural control. This could free up visual processing resources that are simultaneously demanded by the planning and execution of typing the required sequence of key presses.

In summary, the literature shows that light touch can facilitate a posture-cognition dual task by providing a reference signal that assists postural control and therefore lowers the overall workload of the task combination. With respect to assisted typing techniques, it could be argued that light touch provided by an inanimate object, or by the backseat of the chair, should also be facilitative. Indeed, a successful case has been reported of assisted typing with a mechanical arm facilitating the arm gestures toward the keyboard (Oudin et al., 2007). It should be noted that the robotic arm in that case was developed to counteract movement perseverations and so the touch applied to the arm was far from light. Also, comparing robotic and human assistance showed a prominent superiority of the latter. One property of a light touch applied by a human facilitator in assisted typing is that the touch can be maintained across the postural changes associated with typing (leaning toward and back from the keyboard, lateral torso movements in sympathy with the typing arm’s movement across the keyboard). This allows the touch signal to be both present and lightly modulated by the typist’s torso movements, serving as feedback about the movements. Any system that allows maintaining and modulating a tactile reference in this way should theoretically also reduce the cognitive effort of the sensorimotor control task.

## 5. Sensorimotor deficits in DD

The direct effect of any touch-based assistance in the seated typing task is likely to be on sensorimotor coordination. The etiology of the sensorimotor deficits will not be the same across DDs. Even so, if a touch signal could function as a supplementary sensory aid to postural control, it could contribute to reducing the overall cognitive workload of individuals with a range of DDs with associated sensorimotor deficits. This section summarizes the literature on the sensorimotor deficits that accompany a range of DD, with a particular focus on ASD and DS populations. The goal is to note the impact of these sensorimotor deficits on the seated typing task.

Sensorimotor deficits refer to broad impairments in the integration of sensory information that orientate and control motor tasks (Coll et al., 2020). Children and adolescents with sensorimotor deficits show prominent postural and gait deficits along with difficulty in planning and controlling voluntary movements (Damasio and Maurer, 1978; Webber et al., 2004; Galli et al., 2008, 2011; Torres, 2013; Bieć et al., 2014; Fournier et al., 2014; O’Keefe et al., 2016; Lim et al., 2017; Klotzbier et al., 2020).

For individuals with DS, impaired postural control, and equilibrium, as well as weaknesses in head control and trunk stability, can disrupt the proprioceptive system and hinder sensory integration (Uyanik et al., 2003; Georgescu et al., 2016; Jain et al., 2022). These difficulties in maintaining stable posture and coordinating movements can directly impact the ability to sit comfortably and maintain the necessary balance required for typing. The hypotonicity often experienced by individuals with DS may further compound these challenges, making it more difficult to maintain an upright sitting position and stabilize the arms for precise typing gestures.

Similarly, the ASD literature has recently seen the emergence of a motor perspective (Torres and Donnellan, 2015) that acknowledges not only the significant presence of sensorimotor coordination deficits, but also that these might be core features in the characterization of ASD. Individuals with ASD frequently experience sensorimotor coordination deficits, including postural instability, poor task-oriented coordination, and movement planning difficulties (Frith et al., 2003; Mari et al., 2003; Fournier et al., 2014; Arabameri and Sotoodeh, 2015; Mache and Todd, 2016; Lim et al., 2017; Begum Ali et al., 2020). These deficits can affect their ability to sit with stability and perform the coordinated arm movements required for typing. Difficulties in sensory integration, such as integrating visual, vestibular, and proprioceptive information, further contribute to the challenges faced by individuals with ASD when engaging in tasks that demand postural balance and precise arm control (Memari et al., 2013; Doumas et al., 2016). For present purposes, it is noteworthy that cognitive performance and muscle strength have been shown to be related in people with ASD, with higher muscular strength associated with increased cognitive performance (Ludyga et al., 2021).

The seemingly simple task of sitting and typing can pose significant challenges for individuals with DD such as DS or ASD. With regard to DS and ASD, the vast majority of postural studies has focused on impairments in standing balance and gait rather than sitting. Most of the research focusing on sitting while balancing has been done on infants and children with DS and ASD. Broadly, the results show delayed acquisition of the sitting stance and lack of balance control associated with the sitting position in both populations (Connolly and Michael, 1986; Lauteslager et al., 1998; Minshew et al., 2004; Czermainski et al., 2014; Semrud-Clikeman et al., 2014; Arabameri and Sotoodeh, 2015; Marchal et al., 2016; Leezenbaum and Iverson, 2019; Jain et al., 2022).

Maintaining an upright seated position may be an easier task than standing or walking, but it still requires the integration of sensorimotor information (Genthon and Rougier, 2006; Serra-AÑó et al., 2015). Moreover, typing while sitting significantly perturbs upright posture by requiring the typing arm to repeatedly cross the midline, and visual fixation to continuously shift around the keyboard. Thus, the sensorimotor deficits in DS and ASD also impact the perturbed sitting posture required by a typing task (Tsimaras and Fotiadou, 2004; Salar et al., 2014). Trunk stability is an essential component seated balancing (Genthon and Rougier, 2006; Roberts and Vette, 2019). The absence of proprioceptive information from legs and ankles joints makes the trunk primarily responsible of maintaining a balanced upright position (Genthon and Rougier, 2006). The literature indicates that both the DS and ASD populations may have weakened trunk muscular strength, challenging balance while sitting (Kohen-Raz, 1981; Weiss et al., 2013; Salar et al., 2014; Ghaeeni et al., 2015; Aly and Abonour, 2016; Salar and Daneshmandi, 2016; Jain et al., 2022).

Postural and motor coordination deficits in DD are generally associated with disrupted sensorimotor integration which results in difficulties when sensory information load increases (Uyanik et al., 2003; Memari et al., 2013; Doumas et al., 2016; Georgescu et al., 2016; Mache and Todd, 2016; O’Keefe et al., 2016). Difficulties in these domains have adverse consequences in everyday life that affect the execution of daily activities, the development of motor and social skills, and, ultimately, independent functioning (Memari et al., 2013; Lim et al., 2017).

As in the case of EF (see Section “6. EF deficits in DD”), it is important to note that similar sensorimotor deficits may have different etiology in different conditions. In the case of ASD, sensorimotor deficits have been attributed to a general disruption in sensorimotor integration due to cerebellar problems and reduced Purkinje cell numbers (Doumas et al., 2016), impaired cerebellum and basal ganglia (Memari et al., 2013), or dysfunctions of multi synaptic pathways in the brain (Molloy et al., 2003). Regarding DS, sensorimotor deficits are generally attributed to hypotonia, ligament laxity and inherent musculoskeletal characteristics (Galli et al., 2008; Wang et al., 2012; Bieć et al., 2014; Klotzbier et al., 2020). According to some researchers, hypotonicity may disrupt feedback loops and affect the voluntary control of muscles (Georgescu et al., 2016). Uyanik et al. (2003) has proposed that sensory integration dysfunction can be due to a reduced number of neural connections in the motor cortex, basal ganglia, and brain stem.

It is worth noting, that sensorimotor deficits have been described in many other DD. Increased body sway and postural instability have also been observed in different task conditions in young adults with Cerebral Palsy (Donker et al., 2008; Sæther et al., 2015) and Williams syndrome (Barozzi et al., 2013). Deficits in static and dynamic balance have also been reported in adults with Fragile X syndrome (O’Keefe et al., 2016). Furthermore, severe postural instability in children with Prader Willies and Ehlers Danlos syndromes have been documented (Galli et al., 2011). The existence of such similar traits in these different DD coincides with the use of assisted typing interventions in all these conditions.

In summary, for individuals with sensorimotor deficits, sitting and typing can be a complex and demanding task as the required coordination overlaps the sensorimotor impairments they experience. Impaired proprioception, weak trunk stability, and disrupted sensory integration highlight the need for tailored interventions to facilitate their engagement in activities requiring sitting and typing.

## 6. EF deficits in DD

As EF represents the core mechanism that directs cognitive resources in higher order mental tasks (Norman and Shallice, 1986; Baddeley, 1992; Royall et al., 2002), the role of EF deficits has received significant attention in the context of a range of DD including ASD and DS (Lanfranchi et al., 2010; Hessl et al., 2019). For example, adolescents with DS show performance deficits in tasks involving working memory, planning, conceptual shifting, inhibition and set-shifting (Lanfranchi et al., 2010). There is an extensive literature on impairment of EF in the ASD population, especially in planning, flexibility, inhibition, auditory and visuospatial working memory, and verbal fluency (Robinson et al., 2009; Czermainski et al., 2014; Kercood et al., 2014). The distribution of cognitive resources (e.g., executive attention) is a key EF, therefore executive dysfunction would impact the allocation of cognitive resources that are limited in those cases where intellectual disability coexists with DD.

The presence of such executive dysfunction in DS, ASD and other DD connects closely with the seated typing task. The cognitive component associated with typing, (i.e., the generation of linguistic content) relies on EFs (see section “2. Task analysis”) and Table 1, where we presented a list of EFs and their role in a typing task. Deficits in planning would limit the strategies available for organizing the content to be typed (narrating an event, answering a question…), deficits in verbal fluency would impair finding the lexical target of a conceptual referent, and working memory impairment would influence typing effectiveness at the sentence and word level. People with executive dysfunctions may find it difficult to maintain the to-be-typed sentence, or even the graphemic string (this is crucial in one-finger typing systems as seen in assisted typing techniques). Similarly, EF dysfunction would also impact the execution of both the postural and focal motor task components of seated typing. These components include keeping the torso upright and stable and programming the sequence of typing movements. The latter requires visuo-spatial orientation to the letter targets on the keyboard and the inverse kinematics required to navigate the finger to the required key.

Executive function (EF) deficits in DD reduce the speed and effectiveness of linguistic content generation. Simultaneously, they impair the ability to stabilize the body and deploy the precise arm movements required for typing. Aside from adversely affecting both task components, reduced EF capacity also limits the ability to manage dual task demands by flexibly allocating and reallocating resources (Mitra, 2004). The net effect would be slowed content generation, delays in motor execution and increased pressure on working memory as the expression of each narrative unit stretches out in time.

It is worth noting again that EF deficits occur in a range of DD (Pennington and Ozonoff, 1996; Daunhauer and Fidler, 2013; Demetriou et al., 2018), but their etiology may be different in conditions such as Fragile X, Cerebral Palsy and Williams syndrome (Pennington and Ozonoff, 1996; Temple and Sanfilippo, 2003; Lanfranchi et al., 2010; Weierink et al., 2013; Hessl et al., 2019; Wotherspoon et al., 2023). In the case of the ASD, EF deficits have been linked to atypical network connectivity between the prefrontal and other cortical regions (Nomi and Uddin, 2015), or to dysfunctional coordination between the frontal lobe and the rest of the brain (Hill, 2004). In DS, it has been proposed that the co-occurrence of obstructive sleep apnoea, and therefore an obstruction of the upper respiratory tract, may contribute to EF impairment as disrupted and fragmented sleep interferes with the maturation of the prefrontal cortex (Joyce et al., 2020).

## 7. General discussion−Sequences of events

This paper sought to present a possible neural pathway by which supportive touch might facilitate the seated typing task performed by individuals with DD. We showed that seated typing requires the coordination of intertwined sensorimotor and EF components. To type while seated, a person first needs the postural basis of a stable upright stance. Postural stability is achieved through the integration of multisensory information (vestibular, somatosensory, and visual). This invokes EF processes and contributes to the overall cognitive load of the typing task. Besides ensuring a stable stance, the postural system plays a facilitative role in the motor planning. Posture control positions the torso in space and in relation to the keys on the keyboard. Computation of the arm trajectories required to type the required key sequences depends on the position and velocity coordinates of the torso. Recent models suggest that multiple coordinate systems are involved in the integration of multisensory information gathered at eye-, head-, and body-centered levels. Even if the coordinates of the target key are computed at a visual level (eye-centered), a body-centered coordinate system is necessary to compute the position of the arm linked to the torso. Also, the usability of an eye-centered coordinate system depends on head stability which in turn is linked to torso stability. Reduced postural stability would increase the dynamic updating of arm trajectory parameters and add to the EF load of the whole task. Postural control therefore plays a crucial facilitative role in the planning and execution of typing actions. Besides planning the arm trajectories, postural control must also anticipate and counteract the perturbations that arise from repeated manual reaching.

In addition to controlling upright posture and coordinating the arm movement sequences, a seated typist also generates linguistic content to be typed. This is a problem-solving task that coordinates ideation and the process of converting ideas into linguistic form and graphemic representations. Together, these tasks are cognitively demanding as they required a constant shift between long-term memory (ideation) and working memory (processing). They also involve inhibition and updating processes that are key aspects of EF.

The typing task’s sensorimotor and cognitive components are inter-linked and so place concurrent demands on the same cognitive resource pool. The challenges of this task are compounded in people with DD as both their sensorimotor coordination and EF are adversely affected. Multisensory integration capacity for postural stability and motor coordination is reduced, which in turn reduces the already lower level of EF capacity available for the linguistic aspects of the task. The summarized literature suggests that an assistive system that could reduce the cognitive load of postural control and motor planning and execution might free up cognitive resources for linguistic ideation and process. The literature also shows that a light external touch that is not load-bearing but provides an external reference signal can benefit postural control similarly to visual information. If external touch is provided at the level of the torso, as a hand on the shoulder, for example, it can facilitate the stabilization of upright stance and free up visual processing resources. These resources can then be used in coordinating the typing task where visual fixation is involved in planning reaching movements of the arm, locating the symbols on the keys, and matching these to the contents of the orthographic buffer. If the external touch is provided through holding the arm, assistance for posture control may be greater as holding the hand could also counteract the postural perturbations that result from arm movement. However, this clearly increases the possibility of influence from the assistant. Other techniques like hand-over-hand assistance (Reichow, 2013), while clearly different in their goals from assisted typing techniques, may also exploit the reduction of cognitive and sensorimotor workload from the availability of touch. Indeed, if we are less concerned about the autonomy of content production and more interested in helping challenged individuals develop some literacy and typing skills, the possibilities of harnessing touch support can be seen in a different light.

This paper’s argument is not that all the effects of touch in assisted typing are in the form of cognitive load reduction. The existence of the presented pathway for cognitive load reduction does not negate the possibility of specific cuing by the facilitator’s touch. What it does is show that specific cuing is not the only plausible effect of touch on typing task performance. As noted in the outset, the arguments offered in the present paper are developed from the position that assisted typing is a co-created process. Where an individual with DD who has cognitive and sensorimotor deficits is physically assisted, either by touching the shoulder or the arm, it should not be surprising that the facilitator has an influence on the action movements. A motor-impaired patient being physically assisted to walk would not produce movement that is free of the facilitator’s influence. The patient’s gait parameters and trajectory pattern would vary as the facilitator changed, and any kinetic-kinematic analysis would reveal the significant extent of the facilitator’s contribution. Although such locomotion would not be independent motor output, the process of assisted walking could have significant learning and rehabilitative benefits. This would be true even if fully independent walking was never recovered, so long as the assistance provided enabled the patient to make some contribution to their movement.

This important point is clarified by an analogy drawn in Wertsch’s (1984) commentary on Vygotsky’s (1978) concept of the zone of proximal development. Vygotsky’s (1978) concept refers to actions that a learner can currently perform only with teachers’ or more advanced peers’ assistance. Such assistance can scaffold the learner’s development, but only if it has certain key characteristics beyond producing the outputs in question. Wertsch (1984) used the example of a child being helped by an adult to divide 124 by 23. If the adult guides the child using leading questions such as “how many times will 23 go into 124?” and “what do we do with the remainder?,” and so on, their assistance would be of a profoundly different kind to that of another adult who tells the child to write specific numbers in specific locations on the paper. The outcome in both cases would be that the child writes the correct answer, but only the former type of assistance would have served as a scaffold for learning, and therefore been of developmental value. The key point is whether the assistance enabled the learner to have a meaningful role in text generation that they alone could not have had.

The two types of assistance discussed by Wertsch (1984) are important to how we understand assisted typing techniques. If touch only served to provide specific cuing, then the situation would correspond to Wertsch’s (1984) second scenario. The individual with DD would be a conduit for the facilitator’s expression. On the other hand, if touch reduced the cognitive load of even a partial contribution from the individual with DD, the effects on the person’s development, quality of life, connection to carers and sense of self-worth could be impacted positively. Whether the individual could generate, or develop to generate, typed text independently would not be the sole determinant of the value of practising and better understanding touch-assisted typing.

## Data availability statement

The original contributions presented in this study are included in the article/supplementary material, further inquiries can be directed to the corresponding author.

## Author contributions

GN, SM, and GP wrote the first draft of the manuscript. GN, SM, GP, AG, and AE revised the manuscript and contributed to the conceptual planning of the manuscript. All authors contributed to the article and approved the submitted version.

## References

[B1] Abou Khalil G. Doré-Mazars K. Senot P. Wang D. P. Legrand A. (2020). Is it better to sit down, stand up or walk when performing memory and arithmetic activities? *Exp. Brain Res.* 238 2487–2496. 10.1007/s00221-020-05858-z 32851460

[B2] Adams J. A. (1971). A closed-loop theory of motor learning. *J. Mot. Behav.* 3 111–150. 10.1080/00222895.1971.10734898 15155169

[B3] Adkin A. L. Campbell A. D. Chua R. Carpenter M. G. (2008). The influence of postural threat on the cortical response to unpredictable and predictable postural perturbations. *Neurosci. Lett.* 435 120–125. 10.1016/j.neulet.2008.02.018 18337005

[B4] Alesi M. Battaglia G. (2019). “Chapter six - motor development and down syndrome,” in *International review of research in developmental disabilities*, ed. Lanfranchi S. (Cambridge, MA: Academic Press), 169–211. 10.1016/bs.irrdd.2019.06.007

[B5] Allum J. H. J. Keshner E. A. (1984). “Vestibular and proprioceptive control of sway stabilization,” in *Disorders of posture and gait*, eds Bles W. Brandt T. (Amsterdam: Elsevier), 19–40.

[B6] Aly S. Abonour A. (2016). Effect of core stability exercise on postural stability in children with down syndrome. *Int. J. Med. Res. Health Sci.* 5 213–222.

[B7] Al-Yahya E. Dawes H. Smith L. Dennis A. Howells K. Cockburn J. (2011). Cognitive motor interference while walking: A systematic review and meta-analysis. *Neurosci. Biobehav. Rev.* 35 715–728. 10.1016/j.neubiorev.2010.08.008 20833198

[B8] Amboni M. Barone P. Hausdorff J. M. (2013). Cognitive contributions to gait and falls: Evidence and implications. *Mov. Disord.* 28 1520–1533. 10.1002/mds.25674 24132840 PMC4119872

[B9] Andersen G. J. Dyre B. P. (1989). Spatial orientation from optic flow in the central visual field. *Percept. Psychophys.* 45 453–458. 10.3758/BF03210719 2726408

[B10] Andersson G. Hagman J. Talianzadeh R. Svedberg A. Larsen H. C. (2002). Effect of cognitive load on postural control. *Brain Res. Bull.* 58 135–139. 10.1016/S0361-9230(02)00770-0 12121823

[B11] Andersson G. Yardley L. Luxon L. (1998). A dual-task study of interference between mental activity and control of balance. *Am. J. Otol.* 19 632–637. 9752972

[B12] Arabameri E. Sotoodeh M. S. (2015). Early developmental delay in children with autism: A study from a developing country. *Infant Behav. Dev.* 39 118–123. 10.1016/j.infbeh.2015.02.017 25827390

[B13] Aruin A. S. Latash M. L. (1995). The role of motor action in anticipatory postural adjustments studied with self-induced and externally triggered perturbations. *Exp. Brain Res.* 106 291–300. 10.1007/BF00241125 8566194

[B14] Aruin A. S. Latash M. L. (1996). Anticipatory postural adjustments during self-initiated perturbations of different magnitude triggered by a standard motor action. *Electroencephalogr. Clin. Neurophysiol.* 101 497–503. 10.1016/s0013-4694(96)95219-4 9020822

[B15] Baddeley A. (1992). Working memory. *Science* 255 556–559. 10.1126/science.1736359 1736359

[B16] Baddeley A. (1996). Exploring the central executive. *Q. J. Exp. Psychol. Sec. A* 49 5–28. 10.1080/713755608

[B17] Bahureksa L. Najafi B. Saleh A. Sabbagh M. Coon D. Mohler M. J. (2017). The impact of mild cognitive impairment on gait and balance: A systematic review and meta-analysis of studies using instrumented assessment. *Gerontology* 63 67–83. 10.1159/000445831 27172932 PMC5107359

[B18] Baker J. Castro A. Dunn A. K. Mitra S. (2018). Asymmetric interference between cognitive task components and concurrent sensorimotor coordination. *J. Neurophysiol*. 120, 330–342. 10.1152/jn.00073.2018 29641311

[B19] Baldan A. M. S. Alouche S. R. Araujo I. M. G. Freitas S. M. S. F. (2014). Effect of light touch on postural sway in individuals with balance problems: A systematic review. *Gait Posture* 40 1–10. 10.1016/j.gaitpost.2013.12.028 24674637

[B20] Barozzi S. Soi D. Gagliardi C. Selicorni A. Bedeschi M. F. Forti S. (2013). Balance function in patients with Williams syndrome. *Gait Posture* 38 221–225. 10.1016/j.gaitpost.2012.11.012 23219788

[B21] Bayot M. Dujardin K. Tard C. Defebvre L. Bonnet C. T. Allart E. (2018). The interaction between cognition and motor control: A theoretical framework for dual-task interference effects on posture, gait initiation, gait and turning. *Neurophysiol. Clin.* 48 361–375. 10.1016/j.neucli.2018.10.003 30487064

[B22] Beals K. P. (2022). Why we should not presume competence and reframe facilitated communication: A critique of Heyworth, Chan & Lawson. *Evid. Based Commun. Assess. Interv.* 16 66–76. 10.1080/17489539.2022.2097872

[B23] Begum Ali J. Charman T. Johnson M. H. Jones E. J. H. the BASIS/STAARS Team (2020). Early motor differences in infants at elevated likelihood of autism spectrum disorder and/or attention deficit hyperactivity disorder. *J. Autism. Dev. Disord.* 50, 4367–4384. 10.1007/s10803-020-04489-1 32328858 PMC7677154

[B24] Belen’kiĭ V. E. Gurfinkel’ V. S. Pal’tsev E. I. (1967). [Control elements of voluntary movements]. *Biofizika* 12 135–141. 5623488

[B25] Berninger V. W. (1999). Coordinating transcription and text generation in working memory during composing: Automatic and constructive processes. *Learn. Disabil. Q.* 22 99–112. 10.2307/1511269

[B26] Betti S. Castiello U. Begliomini C. (2021). Reach-to-grasp: A multisensory experience. *Front. Psychol.* 12:614471. 10.3389/fpsyg.2021.614471 33633644 PMC7900505

[B27] Bieć E. Zima J. Wójtowicz D. Wojciechowska-Maszkowska B. Krȩcisz K. Kuczyński M. (2014). Postural stability in young adults with down syndrome in challenging conditions. *PLoS One* 9:e94247. 10.1371/journal.pone.0094247 24728178 PMC3984118

[B28] Bolton D. A. E. (2015). The role of the cerebral cortex in postural responses to externally induced perturbations. *Neurosci. Biobehav. Rev.* 57 142–155. 10.1016/j.neubiorev.2015.08.014 26321589

[B29] Bosco A. Piserchia V. Fattori P. (2017). Multiple coordinate systems and motor strategies for reaching movements when eye and hand are dissociated in depth and direction. *Front. Hum. Neurosci.* 11:323. 10.3389/fnhum.2017.00323 28690504 PMC5481402

[B30] Bouisset S. Zattara M. (1988). “Anticipatory postural adjustments and dynamic asymmetry of voluntary movement,” in *Stance and motion: Facts and concepts*, eds Gurfinkel V. S. Ioffe M. E. Massion J. Roll J. P. (Boston, MA: Springer), 177–183. 10.1007/978-1-4899-0821-6_16

[B31] Broadbent D. E. (1958). *Perception and communication.* London: Pergamon Press.

[B32] Brown L. A. Shumway-Cook A. Woollacott M. H. (1999). Attentional demands and postural recovery: The effects of aging. *J. Gerontol. A. Biol. Sci. Med. Sci.* 54 M165–M171. 10.1093/gerona/54.4.M165 10219006

[B33] Brown S. W. (1998). Automaticity versus timesharing in timing and tracking dual-task performance. *Psychol. Res.* 61 71–81. 10.1007/s004260050014

[B34] Bucci M. P. Doyen C. Contenjean Y. Kaye K. (2013). The effect of performing a dual task on postural control in children with autism. *ISRN Neurosci.* 2013:796174. 10.1155/2013/796174 24959567 PMC4045564

[B35] Bullock D. Grossberg S. Guenther F. H. (1993). A self-organizing neural model of motor equivalent reaching and tool use by a multijoint arm. *J. Cogn. Neurosci.* 5 408–435. 10.1162/jocn.1993.5.4.408 23964916

[B36] Buracchio T. J. Mattek N. C. Dodge H. H. Hayes T. L. Pavel M. Howieson D. B. (2011). Executive function predicts risk of falls in older adults without balance impairment. *BMC Geriatr.* 11:74. 10.1186/1471-2318-11-74 22070602 PMC3226437

[B37] Burgess C. A. Kirsch I. Shane H. Niederauer K. L. Graham S. M. Bacon A. (1998). Facilitated communication as an ideomotor response. *Psychol. Sci.* 9 71–74. 10.1111/1467-9280.00013

[B38] Carpenter W. B. (1875). *Principles of mental physiology: With their applications to the training and discipline of the mind, and the study of its morbid conditions.* London: H.S. King and Company.

[B39] Chang C.-H. Wade M. G. Stoffregen T. A. Hsu C.-Y. Pan C.-Y. (2010). Visual tasks and postural sway in children with and without autism spectrum disorders. *Res. Dev. Disabil.* 31 1536–1542. 10.1016/j.ridd.2010.06.003 20598853

[B40] Chen F.-C. Tsai C.-L. (2015). The mechanisms of the effect of light finger touch on postural control. *Neurosci. Lett.* 605 69–73. 10.1016/j.neulet.2015.08.016 26291485

[B41] Chen F.-C. Chen H.-L. Tu J.-H. Tsai C.-L. (2015). Effects of light touch on postural sway and visual search accuracy: A test of functional integration and resource competition hypotheses. *Gait Posture* 42 280–284. 10.1016/j.gaitpost.2015.06.001 26112777

[B42] Chen F.-C. Li L.-L. Chu C.-H. Pan C.-Y. Tsai C.-L. (2019). Finger soaking enhances effects of light touch on reducing body sway in children with developmental coordination disorder. *J. Rehabil. Med.* 51 217–224. 10.2340/16501977-2524 30815705

[B43] Clarys D. Bugaiska A. Tapia G. Baudouin A. (2009). Ageing, remembering, and executive function. *Memory* 17 158–168. 10.1080/09658210802188301 18615347

[B44] Coll S.-M. Foster N. E. V. Meilleur A. Brambati S. M. Hyde K. L. (2020). Sensorimotor skills in autism spectrum disorder: A meta-analysis. *Res. Autism Spectr. Disord.* 76 101570. 10.1016/j.rasd.2020.101570

[B45] Collins J. J. De Luca C. J. (1993). Open-loop and closed-loop control of posture: A random-walk analysis of center-of-pressure trajectories. *Exp. Brain Res.* 95 308–318. 10.1007/BF00229788 8224055

[B46] Connolly B. H. Michael B. T. (1986). Performance of retarded children, with and without down syndrome, on the Bruininks Oseretsky test of motor proficiency. *Phys. Ther.* 66 344–348. 10.1093/ptj/66.3.344 2937069

[B47] Cornoldi C. Del Prete F. Gallani A. Sella F. Re A. M. (2010). “Components affecting expressive writing in typical and disabled writers,” in *Advances in learning and behavioral disabilities*, eds Scruggs T. E. Mastropieri M. A. (Bingley: Emerald Group Publishing Limited), 269–286. 10.1108/S0735-004X20100000023012

[B48] Cummins D. Myer K. Studenka B. (2015). “Hysteresis and motor planning in children with autism spectrum disorder,” in *Proceedings of the 2015 Annual meeting of the North American Society for the Psychology of Sport and Physical Activity (NASPSPA)*, (Portland).

[B49] Curioni A. Knoblich G. Sebanz N. Goswami A. Vadakkepat P. (2019). “Joint action in humans: A model for human-robot interactions,” in *Humanoid robotics: A reference*, eds Goswami A. Vadakkepat P. (Dordrecht: Springer), 2149–2167.

[B50] Czermainski F. R. dos Santos Riesgo R. Guimarães L. S. P. de Salles J. F. Bosa C. A. (2014). Executive functions in children and adolescents with autism spectrum disorder. *Paidéia* 24 85–94. 10.1590/1982-43272457201411

[B51] D’Esposito M. Postle B. R. Ballard D. Lease J. (1999). Maintenance versus manipulation of information held in working memory: An event-related fMRI study. *Brain Cogn.* 41 66–86. 10.1006/brcg.1999.1096 10536086

[B52] Damasio A. R. Maurer R. G. (1978). A neurological model for childhood autism. *Arch. Neurol.* 35 777–786. 10.1001/archneur.1978.00500360001001 718482

[B53] Dault M. C. Frank J. S. Allard F. (2001). Influence of a visuo-spatial, verbal and central executive working memory task on postural control. *Gait Posture* 14 110–116. 10.1016/S0966-6362(01)00113-8 11544062

[B54] Daunhauer L. A. Fidler D. J. (2013). “Executive functioning in individuals with Down syndrome,” in *Handbook of self-regulatory processes in development: New directions and international perspectives*, (New York, NY: Psychology Press), 453–472. 10.4324/9780203080719.ch20

[B55] De Vita F. Schmidt S. Tinti C. Re A. M. (2021). The role of working memory on writing processes. *Front. Psychol.* 12:738395. 10.3389/fpsyg.2021.738395 34512490 PMC8430238

[B56] Demetriou E. A. DeMayo M. M. Guastella A. J. (2019). Executive function in autism spectrum disorder: History, theoretical models, empirical findings, and potential as an endophenotype. *Front. Psychiatry* 10:753. 10.3389/fpsyt.2019.00753 31780959 PMC6859507

[B57] Demetriou E. A. Lampit A. Quintana D. S. Naismith S. L. Song Y. J. C. Pye J. E. (2018). Autism spectrum disorders: A meta-analysis of executive function. *Mol. Psychiatry* 23 1198–1204. 10.1038/mp.2017.75 28439105 PMC5984099

[B58] Diamond A. (2013). Executive functions. *Annu. Rev. Psychol.* 64 135–168. 10.1146/annurev-psych-113011-143750 23020641 PMC4084861

[B59] Diener H. C. Dichgans J. Guschlbauer B. Mau H. (1984). The significance of proprioception on postural stabilization as assessed by ischemia. *Brain Res.* 296 103–109. 10.1016/0006-8993(84)90515-8 6713202

[B60] Diener H. C. Horak F. B. Nashner L. M. (1988). Influence of stimulus parameters on human postural responses. *J. Neurophysiol.* 59 1888–1905. 10.1152/jn.1988.59.6.1888 3404210

[B61] Dijkstra T. M. H. Gielen C. C. A. M. Melis B. J. M. (1992). Postural responses to stationary and moving scenes as a function of distance to the scene. *Hum. Mov. Sci.* 11 195–203. 10.1016/0167-9457(92)90060-O

[B62] Ditthaphongphakdee S. Gaogasigam C. (2020). The effects of light touch cue on gait initiation in patients with Parkinson’s disease. *J. Bodyw. Mov. Ther.* 26 187–192. 10.1016/j.jbmt.2020.08.009 33992243

[B63] Donker S. F. Ledebt A. Roerdink M. Savelsbergh G. J. P. Beek P. J. (2008). Children with cerebral palsy exhibit greater and more regular postural sway than typically developing children. *Exp. Brain Res.* 184 363–370. 10.1007/s00221-007-1105-y 17909773 PMC2137946

[B64] Doumas M. McKenna R. Murphy B. (2016). Postural control deficits in autism spectrum disorder: The role of sensory integration. *J. Autism Dev. Disord.* 46 853–861. 10.1007/s10803-015-2621-4 26446773

[B65] Edwards A. S. (1946). Body sway and vision. *J. Exp. Psychol.* 36 526–535. 10.1037/h0059909 20279299

[B66] Ellis A. (1982). “Spelling and writing (and reading and speaking),” in *Normality and pathology in cognitive functions*, ed. Ellis A. W. (London: Academic Press).

[B67] Emerson A. (2010). “Analyse der bei FC verwendeten Wörter als Indikator für Autorenschaft und Einflussnahme bei der Gestützten Kommunikation,” in *Facilitated Communication. Forschung und Praxis im Dialog*, eds Alfaré A. Huber-Kaiser T. Janz F. Klauß T. (Karlsruhe: von Loeper), 44–50.

[B68] Estevan I. Gandia S. Villarrasa-Sapina I. Bermejo J. L. García-Masso X. (2018). Working memory task influence in postural stability and cognitive function in adolescents. *Motor Control* 22 425–435. 10.1123/mc.2017-0063 29486627

[B69] Fernandes Â Coelho T. Vitória A. Ferreira A. Santos R. Rocha N. (2015). Standing balance in individuals with Parkinson’s disease during single and dual-task conditions. *Gait Posture* 42 323–328. 10.1016/j.gaitpost.2015.06.188 26149283

[B70] Fisk J. E. Sharp C. A. (2004). Age-related impairment in executive functioning: Updating, inhibition, shifting, and access. *J. Clin. Exp. Neuropsychol.* 26 874–890. 10.1080/13803390490510680 15742539

[B71] Fournier K. A. Amano S. Radonovich K. J. Bleser T. M. Hass C. J. (2014). Decreased dynamical complexity during quiet stance in children with autism spectrum disorders. *Gait Posture* 39 420–423. 10.1016/j.gaitpost.2013.08.016 24055002

[B72] Fraizer E. V. Mitra S. (2008a). Methodological and interpretive issues in posture-cognition dual-tasking in upright stance. *Gait Posture* 27 271–279. 10.1016/j.gaitpost.2007.04.002 17524648

[B73] Fraizer E. V. Mitra S. (2008b). Postural costs of performing cognitive tasks in non-coincident reference frames. *Exp. Brain Res.* 185 429–441. 10.1007/s00221-007-1163-1 17960369

[B74] Frith U. Hill E. L. Mari M. Castiello U. Marks D. Marraffa C. (2003). The reach to grasp movement in children with autism spectrum disorder. *Philos. Trans. R. Soc. Lond. B Biol. Sci*. 358, 393–403. 10.1098/rstb.2002.1205 12639336 PMC1693116

[B75] Galli M. Cimolin V. Vismara L. Grugni G. Camerota F. Celletti C. (2011). The effects of muscle hypotonia and weakness on balance: A study on Prader-Willi and Ehlers-Danlos syndrome patients. *Res. Dev. Disabil.* 32 1117–1121. 10.1016/j.ridd.2011.01.015 21306869

[B76] Galli M. Rigoldi C. Mainardi L. Tenore N. Onorati P. Albertini G. (2008). Postural control in patients with down syndrome. *Disabil. Rehabil.* 30 1274–1278. 10.1080/09638280701610353 17943512

[B77] Gazes Y. Rakitin B. C. Steffener J. Habeck C. Butterfield B. Ghez C. (2010). Performance degradation and altered cerebral activation during dual performance: Evidence for a bottom-up attentional system. *Behav. Brain Res.* 210 229–239. 10.1016/j.bbr.2010.02.036 20188768 PMC3531229

[B78] Genthon N. Rougier P. (2006). Does the capacity to appropriately stabilize trunk movements facilitate the control of upright standing? *Motor Control* 10 232–243. 10.1123/mcj.10.3.232 17106132

[B79] Georgescu M. Cernea M. Balan V. (2016). “Postural control in down syndrome subjects,” in *Proceedings of the 5th International Congress of Physical Education, Sports and Kinetotherapy, European Proceedings of Social and Behavioural Sciences*, Vol. 11 eds Grigore V. Stanescu M. Paunescu M. (London: Future Academy), 258–265. 10.15405/epsbs.2016.06.35

[B80] Ghaeeni S. Bahari Z. Khazaei A. A. (2015). Effect of core stability training on static balance of the children with down syndrome. *Phys. Treat. Specif. Phys. Ther. J.* 5 49–54.

[B81] Ghosn N. J. Palmer J. A. Borich M. R. Ting L. H. Payne A. M. (2020). Cortical beta oscillatory activity evoked during reactive balance recovery scales with perturbation difficulty and individual balance ability. *Brain Sci.* 10:860. 10.3390/brainsci10110860 33207570 PMC7697848

[B82] Grayson A. Emerson A. Howard-Jones P. O’Neill L. (2012). Hidden communicative competence: Case study evidence using eye-tracking and video analysis. *Autism Int. J. Res. Pract.* 16 80–95. 10.1177/1362361310393260 21527449

[B83] Hayes J. Flower L. (1980). “Identifying the organization of writing processes,” in *Cognitive Processes in Writing*, eds Gregg L. W. Steinberg E. R. (Hillsdale, NJ: Lawrence Erlbaum), 3–30.

[B84] Hessl D. Schweitzer J. B. Nguyen D. V. McLennan Y. A. Johnston C. Shickman R. (2019). Cognitive training for children and adolescents with fragile X syndrome: A randomized controlled trial of Cogmed. *J. Neurodev. Disord.* 11:4. 10.1186/s11689-019-9264-2 30982467 PMC6463634

[B85] Hill E. (2004). Evaluating the theory of executive dysfunction in autism. *Dev. Rev.* 24 189–233. 10.1016/j.dr.2004.01.001

[B86] Hoff B. Arbib M. A. (1993). Models of trajectory formation and temporal interaction of reach and grasp. *J. Mot. Behav.* 25 175–192. 10.1080/00222895.1993.9942048 12581988

[B87] Holden M. Ventura J. Lackner J. R. (1994). Stabilization of posture by precision contact of the index finger. *J. Vestib. Res.* 4 285–301. 7921347

[B88] Hollands M. Hollands K. Rietdyk S. (2017). “Visual control of adaptive locomotion and changes due to natural ageing,” in *Locomotion and posture in older adults: The role of aging and movement disorders*, eds Barbieri F. A. Vitório R. (Cham: Springer International Publishing), 55–72. 10.1007/978-3-319-48980-3_5

[B89] Horak F. B. Shupert C. L. Dietz V. Horstmann G. (1994). Vestibular and somatosensory contributions to responses to head and body displacements in stance. *Exp. Brain Res.* 100 93–106. 10.1007/BF00227282 7813657

[B90] Hsiu-Chen C. Chiung-Chu C. Jiunn-Woei L. Wei-Da C. Yi-Hsin W. Ya-Ju C. (2020). The effects of dual-task in patients with Parkinson’s disease performing cognitive-motor paradigms. *J. Clin. Neurosci.* 72 72–78. 10.1016/j.jocn.2020.01.024 31952973

[B91] Isreal J. B. Chesney G. L. Wickens C. D. Donchin E. (1980). P300 and tracking difficulty: Evidence for multiple resources in dual-task performance. *Psychophysiology* 17 259–273. 10.1111/j.1469-8986.1980.tb00146.x 7384376

[B92] Jacobs J. V. (2014). Why we need to better understand the cortical neurophysiology of impaired postural responses with age, disease, or injury. *Front. Integr. Neurosci.* 8:69. 10.3389/fnint.2014.00069 25221483 PMC4148624

[B93] Jacobs J. V. Horak F. B. (2007). Cortical control of postural responses. *J. Neural Transm.* 114 1339–1348. 10.1007/s00702-007-0657-0 17393068 PMC4382099

[B94] Jain P. D. Nayak A. Karnad S. D. (2022). Relationship between trunk muscle strength, reaching ability and balance in children with down syndrome - A cross-sectional study. *Brain Dev.* 44 95–104. 10.1016/j.braindev.2021.09.005 34579982

[B95] Jaswal V. K. Wayne A. Golino H. (2020). Eye-tracking reveals agency in assisted autistic communication. *Sci. Rep.* 10:7882. 10.1038/s41598-020-64553-9 32398782 PMC7217901

[B96] Jeka J. J. (1997). Light touch contact as a balance aid. *Phys. Ther.* 77 476–487. 10.1093/ptj/77.5.476 9149759

[B97] Jeka J. J. Lackner J. R. (1994). Fingertip contact influences human postural control. *Exp. Brain Res.* 100 495–502. 10.1007/BF02738408 7813685

[B98] Jeka J. J. Lackner J. R. (1995). The role of haptic cues from rough and slippery surfaces in human postural control. *Exp. Brain Res.* 103 267–276. 10.1007/BF00231713 7789434

[B99] Jeka J. J. Easton R. D. Bentzen B. L. Lackner J. R. (1996). Haptic cues for orientation and postural control. *Percept. Psychophys.* 58 409–423. 10.3758/BF03206817 8935902

[B100] Johannsen L. Guzman-Garcia A. Wing A. M. (2009). Interpersonal light touch assists balance in the elderly. *J. Mot. Behav.* 41 397–399. 10.3200/35-09-001 19460746

[B101] Johannsen L. Wing A. M. Hatzitaki V. (2011). Contrasting effects of finger and shoulder interpersonal light touch on standing balance. *J. Neurophysiol.* 107 216–225. 10.1152/jn.00149.2011 21957227

[B102] Joyce A. Elphick H. Farquhar M. Gringras P. Evans H. Bucks R. S. (2020). Obstructive sleep apnoea contributes to executive function impairment in young children with down syndrome. *Behav. Sleep Med.* 18 611–621. 10.1080/15402002.2019.1641501 31311334

[B103] Kaulmann D. Saveriano M. Lee D. Hermsdörfer J. Johannsen L. (2020). Stabilization of body balance with light touch following a mechanical perturbation: Adaption of sway and disruption of right posterior parietal cortex by cTBS. *PLoS One* 15:e0233988. 10.1371/journal.pone.0233988 32615583 PMC7332304

[B104] Kearney F. C. Harwood R. H. Gladman J. R. F. Lincoln N. Masud T. (2013). The relationship between executive function and falls and gait abnormalities in older adults: A systematic review. *Dement. Geriatr. Cogn. Disord.* 36 20–35. 10.1159/000350031 23712088

[B105] Kercood S. Grskovic J. A. Banda D. Begeske J. (2014). Working memory and autism: A review of literature. *Res. Autism Spectr. Disord.* 8 1316–1332. 10.1016/j.rasd.2014.06.011

[B106] Klotzbier T. J. Bühler K. Holfelder B. Schott N. (2020). Exploring motor-cognitive interference in children with down syndrome using the Trail-Walking-Test. *Res. Dev. Disabil.* 106 103769. 10.1016/j.ridd.2020.103769 32979845

[B107] Kohen-Raz R. (1981). Postural control and learning disabilities. *Early Child Dev. Care* 7 329–352. 10.1080/0300443810070406

[B108] Kramer A. F. Wickens C. D. Donchin E. (1983). An analysis of the processing requirements of a complex perceptual-motor task. *Hum. Factors* 25 597–621. 10.1177/001872088302500601 6671646

[B109] Krishnamoorthy V. Slijper H. Latash M. L. (2002). Effects of different types of light touch on postural sway. *Exp. Brain Res.* 147 71–79. 10.1007/s00221-002-1206-6 12373371

[B110] Kuo A. D. (2005). An optimal state estimation model of sensory integration in human postural balance. *J. Neural Eng.* 2 S235–S249. 10.1088/1741-2560/2/3/S07 16135887

[B111] Lajoie Y. Teasdale N. Bard C. Fleury M. (1996). “Attentional demands for walking: Age-related changes,” in *Advances in psychology changes in sensory motor behavior in aging*, eds Ferrandez A.-M. Teasdale N. (Amsterdam: Elsevier), 235–256. 10.1016/S0166-4115(96)80011-2

[B112] Lanfranchi S. Jerman O. Pont E. D. Alberti A. Vianello R. (2010). Executive function in adolescents with down Syndrome. *J. Intellect. Disabil. Res.* 54 308–319. 10.1111/j.1365-2788.2010.01262.x 20202074

[B113] Lauteslager P. Vermeer A. Helders P. (1998). Disturbances in the motor behaviour of children with Down’s syndrome: The need for a theoretical framework. *Physiotherapy* 84 5–13. 10.1016/S0031-9406(05)65896-8

[B114] Lee D. N. Lishman J. R. (1975). Visual proprioceptive control of stance. *J. Hum. Mov. Stud.* 1 87–95.

[B115] Lee Y. Curuk E. Aruin A. S. (2020). Effect of light finger touch, a cognitive task, and vision on standing balance in stroke. *J. Mot. Behav.* 53 157–165. 10.1080/00222895.2020.1742082 32281912

[B116] Lee Y. Goyal N. Aruin A. S. (2018). Effect of a cognitive task and light finger touch on standing balance in healthy adults. *Exp. Brain Res.* 236 399–407. 10.1007/s00221-017-5135-9 29164286

[B117] Leezenbaum N. B. Iverson J. M. (2019). Trajectories of posture development in infants with and without familial risk for autism spectrum disorder. *J. Autism Dev. Disord.* 49 3257–3277. 10.1007/s10803-019-04048-3 31079276

[B118] Li K. Z. H. Lindenberger U. (2002). Relations between aging sensory/sensorimotor and cognitive functions. *Neurosci. Biobehav. Rev.* 26 777–783. 10.1016/S0149-7634(02)00073-8 12470689

[B119] Lilienfeld S. O. Marshall J. Todd J. T. Shane H. C. (2014). The persistence of fad interventions in the face of negative scientific evidence: Facilitated communication for autism as a case example. *Evid. Based Commun. Assess. Interv.* 8 62–101. 10.1080/17489539.2014.976332

[B120] Lim Y. H. Partridge K. Girdler S. Morris S. L. (2017). Standing postural control in individuals with autism spectrum disorder: Systematic review and meta-analysis. *J. Autism Dev. Disord.* 47 2238–2253. 10.1007/s10803-017-3144-y 28508177

[B121] Ludyga S. Pühse U. Gerber M. Mücke M. (2021). Muscle strength and executive function in children and adolescents with autism spectrum disorder. *Autism Res.* 14 2555–2563. 10.1002/aur.2587 34351051 PMC9292567

[B122] Mache M. A. Todd T. A. (2016). Gross motor skills are related to postural stability and age in children with autism spectrum disorder. *Res. Autism Spectr. Disord.* 23 179–187. 10.1016/j.rasd.2016.01.001

[B123] Magalhães F. H. Kohn A. F. (2011). Vibratory noise to the fingertip enhances balance improvement associated with light touch. *Exp. Brain Res.* 209 139–151. 10.1007/s00221-010-2529-3 21191573

[B124] Mahboobin A. Loughlin P. J. Redfern M. S. (2007). A model-based approach to attention and sensory integration in postural control of older adults. *Neurosci. Lett.* 429 147–151. 10.1016/j.neulet.2007.10.004 17997035 PMC2440587

[B125] Maki B. E. Zecevic A. Bateni H. Kirshenbaum N. Mcilroy W. E. (2001). Cognitive demands of executing postural reactions: Does aging impede attention switching? *Neuroreport* 12 3583–3587. 10.1097/00001756-200111160-00042 11733716

[B126] Marchal J. P. Maurice-Stam H. Houtzager B. A. Rutgers Van Rozenburg-Marres S. L. Oostrom K. J. Grootenhuis M. A. (2016). Growing up with Down syndrome: Development from 6 months to 10.7 years. *Res. Dev. Disabil.* 59 437–450. 10.1016/j.ridd.2016.09.019 27744268

[B127] Mari M. Castiello U. Marks D. Marraffa C. Prior M. (2003). The reach-to-grasp movement in children with autism spectrum disorder. *Philos. Trans. R. Soc. Lond. B. Biol. Sci.* 358 393–403. 10.1098/rstb.2002.1205 12639336 PMC1693116

[B128] Martinelli A. R. Coelho D. B. Teixeira L. A. (2018). Light touch leads to increased stability in quiet and perturbed balance: Equivalent effects between post-stroke and healthy older individuals. *Hum. Mov. Sci.* 58 268–278. 10.1016/j.humov.2018.03.001 29524852

[B129] Masson G. Mestre D. R. Pailhous J. (1995). Effects of the spatio-temporal structure of optical flow on postural readjustments in man. *Exp. Brain Res.* 103 137–150. 10.1007/BF00241971 7615029

[B130] McCutchen D. (2000). Knowledge, processing, and working memory: Implications for a theory of writing. *Educ. Psychol.* 35 13–23. 10.1207/S15326985EP3501_3

[B131] Mcilroy W. E. Norrie R. G. Brooke J. D. Bishop D. C. Nelson A. J. Maki B. E. (1999). Temporal properties of attention sharing consequent to disturbed balance. *Neuroreport* 10 2895–2899. 10.1097/00001756-199909290-00004 10549793

[B132] Memari A. H. Ghanouni P. Gharibzadeh S. Eghlidi J. Ziaee V. Moshayedi P. (2013). Postural sway patterns in children with autism spectrum disorder compared with typically developing children. *Res. Autism Spectr. Disord.* 7 325–332. 10.1016/j.rasd.2012.09.010

[B133] Meyer D. E. Abrams R. A. Kornblum S. Wright C. E. Keith Smith J. E. (1988). Optimality in human motor performance: Ideal control of rapid aimed movements. *Psychol. Rev.* 95 340–370. 10.1037/0033-295X.95.3.340 3406245

[B134] Meyer D. E. Kieras D. E. (1997). A computational theory of executive cognitive processes and multiple-task performance: Part 1. Basic mechanisms. *Psychol. Rev.* 104 3–65. 10.1037/0033-295x.104.1.3 9009880

[B135] Miall R. C. Wolpert D. M. (1996). Forward models for physiological motor control. *Neural Netw.* 9 1265–1279. 10.1016/S0893-6080(96)00035-4 12662535

[B136] Minshew N. J. Sung K. Jones B. L. Furman J. M. (2004). Underdevelopment of the postural control system in autism. *Neurology* 63 2056–2061. 10.1212/01.WNL.0000145771.98657.62 15596750

[B137] Mitra S. (2004). Adaptive utilization of optical variables during postural and suprapostural dual-task performance: Comment on Stoffregen, Smart, Bardy, and Pagulayan (1999). *J. Exp. Psychol.* 30 28–38. 10.1037/0096-1523.30.1.28 14769066

[B138] Miyake A. Friedman N. P. (2012). The nature and organization of individual differences in executive functions: Four general conclusions. *Curr. Dir. Psychol. Sci.* 21 8–14. 10.1177/0963721411429458 22773897 PMC3388901

[B139] Miyake A. Friedman N. P. Emerson M. J. Witzki A. H. Howerter A. Wager T. D. (2000). The unity and diversity of executive functions and their contributions to complex “Frontal Lobe” tasks: A latent variable analysis. *Cogn. Psychol.* 41 49–100. 10.1006/cogp.1999.0734 10945922

[B140] Molloy C. A. Dietrich K. N. Bhattacharya A. (2003). Postural stability in children with autism spectrum disorder. *J. Autism Dev. Disord.* 33 643–652. 10.1023/b:jadd.0000006001.00667.4c 14714933

[B141] Moore S. Brunt D. Nesbitt M. L. Juarez T. (1992). Investigation of evidence for anticipatory postural adjustments in seated subjects who performed a reaching task. *Phys. Ther.* 72 335–343. 10.1093/ptj/72.5.335 1631202

[B142] Morasso P. G. Baratto L. Capra R. Spada G. (1999). Internal models in the control of posture. *Neural Netw.* 12 1173–1180. 10.1016/s0893-6080(99)00058-1 12662652

[B143] Morenilla L. Márquez G. Sánchez J. A. Bello O. López-Alonso V. Fernández-Lago H. (2020). Postural stability and cognitive performance of subjects with Parkinson’s disease during a dual-task in an upright stance. *Front. Psychol.* 11:256. 10.3389/fpsyg.2020.01256 32903649 PMC7438725

[B144] Morris M. Iansek R. Smithson F. Huxham F. (2000). Postural instability in Parkinson’s disease: A comparison with and without a concurrent task. *Gait Posture* 12 205–216. 10.1016/s0966-6362(00)00076-x 11154931

[B145] Mostert M. P. (2001). Facilitated communication since 1995: A review of published studies. *J. Autism Dev. Disord.* 31 287–313. 10.1023/a:1010795219886 11518483

[B146] Mostert M. P. (2010). Facilitated communication and its legitimacy—twenty-first century developments. *Exceptionality* 18 31–41. 10.1080/09362830903462524

[B147] Nadkarni N. K. Zabjek K. Lee B. McIlroy W. E. Black S. E. (2010). Effect of working memory and spatial attention tasks on gait in healthy young and older adults. *Mot. Control* 14 195–210. 10.1123/mcj.14.2.195 PMC3897230 20484770

[B148] Navon D. Gopher D. (1979). On the economy of the human-processing system. *Psychol. Rev.* 86 214–255. 10.1037/0033-295X.86.3.214

[B149] NICE (2012). *Recommendations | autism spectrum disorder in adults: Diagnosis and management | Guidance | NICE*. Available online at: https://www.nice.org.uk/guidance/cg142/chapter/Recommendations Paragraph 1.4.3. (accessed July 24, 2023).

[B150] Nicoli G. Mitra S. Pavon G. Grayson A. Emerson A. E. Cortelazzo M. (2021). *Individuals with developmental disabilities do make individual stylistic contributions to text written with physical facilitation.* Available online at: https://osf.io/preprints/socarxiv/m9r62/ (accessed July 20, 2021). 10.3389/frcha.2023.1182884 PMC11731646 39816894

[B151] Nomi J. S. Uddin L. Q. (2015). Developmental changes in large-scale network connectivity in autism. *Neuroimage Clin.* 7 732–741. 10.1016/j.nicl.2015.02.024 25844325 PMC4375789

[B152] Norman D. A. Shallice T. (1986). “Attention to action,” in *Consciousness and self-regulation: Advances in research and theory*, Vol. 4 eds Davidson R. J. Schwartz G. E. Shapiro D. (Boston, MA: Springer), 1–18. 10.1007/978-1-4757-0629-1_1

[B153] Norrie R. G. Maki B. E. Staines W. R. McIlroy W. E. (2002). The time course of attention shifts following perturbation of upright stance. *Exp. Brain Res.* 146 315–321. 10.1007/s00221-002-1172-z 12232688

[B154] O’Keefe J. A. Robertson-Dick E. E. Hall D. A. Berry-Kravis E. (2016). Gait and functional mobility deficits in Fragile X-associated tremor/ataxia syndrome. *Cerebellum* 15 475–482. 10.1007/s12311-015-0714-4 26298472 PMC8797146

[B155] Onofrei R. R. Amaricai E. Suciu O. David V. L. Rata A. L. Hogea E. (2020). Smartphone use and postural balance in healthy young adults. *Int. J. Environ. Res. Public Health* 17:3307. 10.3390/ijerph17093307 32397463 PMC7246876

[B156] Oudin N. Revel A. Nadel J. (2007). Quand une machine facilite l’écriture d’enfants non verbaux avec autisme. *Enfance* 59 82–91.

[B157] Palluel E. Chauvel G. Bourg V. Commare M.-C. Prado C. Farigoule V. (2019). Effects of dual tasking on postural and gait performances in children with cerebral palsy and healthy children. *Int. J. Dev. Neurosci.* 79 54–64. 10.1016/j.ijdevneu.2019.10.008 31722225

[B158] Pashler H. (1994). Dual-task interference in simple tasks: Data and theory. *Psychol. Bull.* 116 220–244. 10.1037/0033-2909.116.2.220 7972591

[B159] Patel P. Lamar M. Bhatt T. (2014). Effect of type of cognitive task and walking speed on cognitive-motor interference during dual-task walking. *Neuroscience* 260 140–148. 10.1016/j.neuroscience.2013.12.016 24345478

[B160] Paulus W. M. Straube A. Brandt T. (1984). Visual stabilization of posture. Physiological stimulus characteristics and clinical aspects. *Brain* 107(Pt. 4) 1143–1163. 10.1093/brain/107.4.1143 6509312

[B161] Paulus W. M. Straube A. Krafczyk S. Brandt T. (1989). Differential effects of retinal target displacement, changing size and changing disparity in the control of anterior/posterior and lateral body sway. *Exp. Brain Res.* 78 243–252. 10.1007/BF00228896 2599035

[B162] Pellecchia G. L. (2003). Postural sway increases with attentional demands of concurrent cognitive task. *Gait Posture* 18 29–34. 10.1016/s0966-6362(02)00138-8 12855298

[B163] Pennington B. F. Ozonoff S. (1996). Executive functions and developmental psychopathology. *J. Child Psychol. Psychiatry* 37 51–87. 10.1111/j.1469-7610.1996.tb01380.x 8655658

[B164] Peper C. E. Oorthuizen J. K. Roerdink M. (2012). Attentional demands of cued walking in healthy young and elderly adults. *Gait Posture* 36 378–382. 10.1016/j.gaitpost.2012.03.032 22608701

[B165] Peterson D. S. Dijkstra B. W. Horak F. B. (2016). Postural motor learning in people with Parkinson’s disease. *J. Neurol.* 263 1518–1529. 10.1007/s00415-016-8158-4 27193311 PMC4972681

[B166] Pisotta I. Molinari M. (2014). Cerebellar contribution to feedforward control of locomotion. *Front. Hum. Neurosci.* 8:475. 10.3389/fnhum.2014.00475 25009490 PMC4069484

[B167] Polit A. Bizzi E. (1979). Characteristics of motor programs underlying arm movements in monkeys. *J. Neurophysiol.* 42 183–194. 10.1152/jn.1979.42.1.183 107279

[B168] Posner M. I. (1980). Orienting of attention. *Q. J. Exp. Psychol.* 32 3–25. 10.1080/00335558008248231 7367577

[B169] Przysucha E. Vollebregt B. Zerpa C. (2020). Impact of attentional loading and task constraints on postural control of healthy older adults. *Int. J. Extreme Autom. Connectiv. Healthc.* 2 12–25. 10.4018/IJEACH.2020070102

[B170] Purcell J. Turkeltaub P. Eden G. Rapp B. (2011). Examining the central and peripheral processes of written word production through meta-analysis. *Front. Psychol.* 2:239. 10.3389/fpsyg.2011.00239 22013427 PMC3190188

[B171] Quant S. Adkin A. L. Staines W. R. Maki B. E. Mcilroy W. E. (2004). The effect of a concurrent cognitive task on cortical potentials evoked by unpredictable balance perturbations. *BMC Neurosci.* 5:18. 10.1186/1471-2202-5-18 15147586 PMC428574

[B172] Rabin E. Chen J. Muratori L. DiFrancisco-Donoghue J. Werner W. G. (2013). Haptic feedback from manual contact improves balance control in people with Parkinson’s disease. *Gait Posture* 38 373–379. 10.1016/j.gaitpost.2012.12.008 23313411 PMC3664138

[B173] Rankin J. Woollacott M. Shumway-Cook A. Brown L. (2000). Cognitive influence on postural stability: A neuromuscular analysis in young and older adults. *J. Gerontol.* 55 M112–M119. 10.1093/gerona/55.3.m112 10795721

[B174] Recarte M. A. Nunes L. M. (2003). Mental workload while driving: Effects on visual search, discrimination, and decision making. *J. Exp. Psychol. Appl.* 9 119–137. 10.1037/1076-898X.9.2.119 12877271

[B175] Reichow B. (2013). “Hand-over-hand assistance,” in *Encyclopedia of autism spectrum disorders*, ed. Volkmar F. R. (New York, NY: Springer), 1483–1484. 10.1007/978-1-4419-1698-3_1159

[B176] Reiser J. E. Wascher E. Rinkenauer G. Arnau S. (2021). Cognitive-motor interference in the wild: Assessing the effects of movement complexity on task switching using mobile EEG. *Eur. J. Neurosci.* 54, 8175–8195. 10.1111/ejn.14959 32889772

[B177] Riccio G. E. Newell K. M. Corcos D. M. (1993). *Variability and motor control.* Champaign, IL: Human Kinetics Publishers.

[B178] Riley M. A. Stoffregen T. A. Grocki M. J. Turvey M. T. (1999). Postural stabilization for the control of touching. *Hum. Mov. Sci.* 18 795–817. 10.1016/S0167-9457(99)00041-X

[B179] Riley M. A. Wong S. Mitra S. Turvey M. T. (1997). Common effects of touch and vision on postural parameters. *Exp. Brain Res.* 117 165–170. 10.1007/s002210050211 9386016

[B180] Roberts B. W. R. Vette A. H. (2019). A kinematics recommendation for trunk stability and control assessments during unstable sitting. *Med. Eng. Phys.* 73 73–76. 10.1016/j.medengphy.2019.08.004 31495723

[B181] Robinson S. Goddard L. Dritschel B. Wisley M. Howlin P. (2009). Executive functions in children with autism spectrum disorders. *Brain Cogn.* 71 362–368. 10.1016/j.bandc.2009.06.007 19628325

[B182] Roll J. P. Roll R. (1988). “From eye to foot: A proprioceptive chain involved in postural control,” in *Posture and gait: Development, adaptation, and modulation*, eds Amblard B. Berthoz A. Clarac F. (Amsterdam: Elsevier), 155–164.

[B183] Romberg M. H. (1853). *Manual of nervous diseases of man.* London: Sydenham Society.

[B184] Royall D. R. Lauterbach E. C. Cummings J. L. Reeve A. Rummans T. A. Kaufer D. I. (2002). Executive control function: A review of its promise and challenges for clinical research. A report from the Committee on Research of the American Neuropsychiatric Association. *J. Neuropsychiatry Clin. Neurosci.* 14 377–405. 10.1176/jnp.14.4.377 12426407

[B185] Sæther R. Helbostad J. L. Adde L. Brændvik S. Lydersen S. Vik T. (2015). The relationship between trunk control in sitting and during gait in children and adolescents with cerebral palsy. *Dev. Med. Child Neurol.* 57 344–350. 10.1111/dmcn.12628 25412837

[B186] Salar S. Daneshmandi H. (2016). The effect of 8 weeks of core stability training program on lumbar-pelvic function in children with autism spectrum. *Sport Sci. Health Res.* 8 67–81. 10.22059/jsmed.2016.58872

[B187] Salar S. Daneshmandi H. Karimizadeh Ardakani M. Nazari Sharif H. (2014). The relationship of core strength with static and dynamic balance in children with autism. *Ann. Appl. Sport Sci.* 2 33–42. 10.18869/acadpub.aassjournal.2.4.33 30186993

[B188] Salas N. Silvente S. (2020). The role of executive functions and transcription skills in writing: A cross-sectional study across 7 years of schooling. *Read Writ.* 33 877–905. 10.1007/s11145-019-09979-y

[B189] Saloviita T. (2018). Does linguistic analysis confirm the validity of facilitated communication? *Focus Autism Other Dev. Disabl.* 33 91–99. 10.1177/1088357616646075

[B190] Saloviita T. Leppänen M. Ojalammi U. (2014). Authorship in facilitated communication: An analysis of 11 cases. *Augment Altern. Commun.* 30 213–225. 10.3109/07434618.2014.927529 24946681

[B191] Saltzman E. Kelso J. A. (1987). Skilled actions: A task-dynamic approach. *Psychol. Rev.* 94 84–106. 10.1037/0033-295X.94.1.84 3823306

[B192] Sartori L. Betti S. (2015). Complementary actions. *Front. Psychol.* 6:557. 10.3389/fpsyg.2015.00557 25983717 PMC4416362

[B193] Schieppati M. Schmid M. Sozzi S. (2014). Rapid processing of haptic cues for postural control in blind subjects. *Clin. Neurophysiol.* 125 1427–1439. 10.1016/j.clinph.2013.11.011 24332472

[B194] Schlosser R. W. Balandin S. Hemsley B. Iacono T. Probst P. von Tetzchner S. (2014). Facilitated communication and authorship: A systematic review. *Augment. Altern. Commun.* 30 359–368. 10.3109/07434618.2014.971490 25384895

[B195] Schütz C. Schack T. (2020). Working memory load does not affect sequential motor planning. *Acta Psychol.* 208:103091. 10.1016/j.actpsy.2020.103091 32485340

[B196] Semrud-Clikeman M. Fine J. G. Bledsoe J. (2014). Comparison among children with children with autism spectrum disorder, nonverbal learning disorder and typically developing children on measures of executive functioning. *J. Autism Dev. Disord.* 44 331–342. 10.1007/s10803-013-1871-2 23812759

[B197] Serra-AÑó P. López-Bueno L. García-Massó X. Pellicer-Chenoll M. T. González L.-M. (2015). Postural control mechanisms in healthy adults in sitting and standing positions. *Percept. Mot. Skills* 121 119–134. 10.2466/26.25.PMS.121c10x4 26108061

[B198] Shadmehr R. Wise S. P. (2005). *The computational neurobiology of reaching and pointing.* Cambridge, MA: MIT press.

[B199] Shallice T. Burgess P. W. (1991). Deficits in strategy application following frontal lobe damage in man. *Brain* 114 727–741. 10.1093/brain/114.2.727 2043945

[B200] Shepherd R. B. Crosbie J. Squires T. (1993). “The contribution of the ipsilateral leg to postural adjustments during fast voluntary reaching in sitting,” in *Paper presented at the of the 14th Congress of the International Society for Biomechanics*, (Paris).

[B201] Shumway-Cook A. Horak F. B. (1986). Assessing the influence of sensory interaction of balance. Suggestion from the field. *Phys. Ther.* 66 1548–1550. 10.1093/ptj/66.10.1548 3763708

[B202] Shumway-Cook A. Woollacott M. (2000). Attentional demands and postural control: The effect of sensory context. *J. Gerontol. A Biol. Sci. Med. Sci.* 55 M10–M16. 10.1093/gerona/55.1.m10 10719767

[B203] Shumway-Cook A. Woollacott M. H. (2012). *Motor control: Translating research into clinical practice.* Philadelphia: Wolters Kluwer Health/Lippincott Williams and Wilkins.

[B204] Shumway-Cook A. Woollacott M. Kerns K. A. Baldwin M. (1997). The effects of two types of cognitive tasks on postural stability in older adults with and without a history of falls. *J. Gerontol. A. Biol. Sci. Med. Sci.* 52A M232–M240. 10.1093/gerona/52A.4.M232 9224435

[B205] Stephenson M. L. Ostrander A. G. Norasi H. Dorneich M. C. (2020). Shoulder muscular fatigue from static posture concurrently reduces cognitive attentional resources. *Hum. Factors* 62 589–602. 10.1177/0018720819852509 31216186

[B206] Stoffregen T. A. Hove P. Bardy B. G. Riley M. Bonnet C. T. (2007). Postural stabilization of perceptual but not cognitive performance. *J. Mot. Behav.* 39 126–138. 10.3200/JMBR.39.2.126-138 17428758

[B207] Stoffregen T. A. Pagulayan R. Bardy B. Hettinger L. (2000). Modulating postural control to facilitate visual performance. *Hum. Mov. Sci.* 19 203–220. 10.1016/S0167-9457(00)00009-9

[B208] Stoffregen T. A. Smart L. J. Bardy B. G. Pagulayan R. J. (1999). Postural stabilization of looking. *J. Exp. Psychol.* 25 1641–1658. 10.1037/0096-1523.25.6.1641

[B209] Strayer D. L. Johnston W. A. (2001). Driven to distraction: Dual-task studies of simulated driving and conversing on a cellular telephone. *Psychol. Sci.* 12 462–466. 10.1111/1467-9280.00386 11760132

[B210] Studenka B. E. Myers K. (2020). Preliminary evidence that motor planning is slower and more difficult for children with autism spectrum disorder during motor cooperation. *Mot. Control* 24 127–149. 10.1123/mc.2019-0007 31369997

[B211] Suzuki M. Miyai I. Ono T. Oda I. Konishi I. Kochiyama T. (2004). Prefrontal and premotor cortices are involved in adapting walking and running speed on the treadmill: An optical imaging study. *Neuroimage* 23 1020–1026. 10.1016/j.neuroimage.2004.07.002 15528102

[B212] Swanenburg J. de Bruin E. D. Uebelhart D. Mulder T. (2009). Compromising postural balance in the elderly. *Gerontology* 55 353–360. 10.1159/000212757 19365104

[B213] Swerdloff M. M. Hargrove L. J. (2020). “Quantifying cognitive load using EEG during ambulation and postural tasks,” in *Proceedings of the 2020 42nd Annual International Conference of the IEEE Engineering in Medicine Biology Society (EMBC)*, (Montreal, QC), 2849–2852. 10.1109/EMBC44109.2020.9176264 33018600

[B214] Temple C. M. Sanfilippo P. M. (2003). Executive skills in Klinefelter’s syndrome. *Neuropsychologia* 41 1547–1559. 10.1016/S0028-3932(03)00061-7 12849773

[B215] Thelen E. Spencer J. P. (1998). Postural control during reaching in young infants: A dynamic systems approach. *Neurosci. Biobehav. Rev.* 22 507–514. 10.1016/S0149-7634(97)00037-7 9595562

[B216] Tombu M. Jolicoeur P. (2003). A central capacity sharing model of dual-task performance. *J. Exp. Psychol. Hum. Percept. Perform.* 29 3–18. 10.1037//0096-1523.29.1.3 12669744

[B217] Torres E. B. (2013). Signatures of movement variability anticipate hand speed according to levels of intent. *Behav. Brain Funct.* 9:10. 10.1186/1744-9081-9-10 23497360 PMC3635904

[B218] Torres E. B. Donnellan A. M. (2015). Editorial for research topic “Autism: The movement perspective.”. *Front. Integr. Neurosci.* 9:12. 10.3389/fnint.2015.00012 25852501 PMC4360560

[B219] Tostanoski A. Lang R. Raulston T. Carnett A. Davis T. (2014). Voices from the past: Comparing the rapid prompting method and facilitated communication. *Dev. Neurorehabil.* 17 219–223. 10.3109/17518423.2012.749952 24102487

[B220] Tresilian J. (2012). *Sensorimotor control and learning: An introduction to the behavioral neuroscience of action.* Basingstoke: Palgrave Macmillan.

[B221] Tsimaras V. K. Fotiadou E. G. (2004). Effect of training on the muscle strength and dynamic balance ability of adults with down syndrome. *J. Strength Cond. Res.* 18 343–347. 10.1519/R-12832.1 15142004

[B222] Tuzzi A. (2009). Grammar and lexicon in individuals with autism: A quantitative analysis of a large Italian corpus. *Intellect. Dev. Disabil.* 47 373–385. 10.1352/1934-9556-47.5.373 19842741

[B223] Uyanik M. Bumin G. Kayilhan H. (2003). Comparison of different therapy approaches in children with down syndrome. *Pediatr. Int.* 45 68–73. 10.1046/j.1442-200X.2003.01670.x 12654073

[B224] Varas-Diaz G. Kannan L. Bhatt T. (2020). Effect of mental fatigue on postural sway in healthy older adults and stroke populations. *Brain Sci.* 10:388. 10.3390/brainsci10060388 32575383 PMC7349503

[B225] Vygotsky L. S. (1978). *Mind in society: Development of higher psychological processes.* Cambridge, MA: Harvard University Press.

[B226] Wang H.-Y. Long I.-M. Liu M.-F. (2012). Relationships between task-oriented postural control and motor ability in children and adolescents with down syndrome. *Res. Dev. Disabil.* 33 1792–1798. 10.1016/j.ridd.2012.05.002 22699252

[B227] Warren W. (2010). “Optic flow,” in *The senses: A comprehensive reference*, eds Masland R. Albright T. D. (Cambridge, MA: Academic Press), 219–230. 10.1016/B978-012370880-9.00311-X

[B228] Webber A. Virji-Babul N. Edwards R. Lesperance M. (2004). Stiffness and postural stability in adults with Down syndrome. *Exp. Brain Res.* 155 450–458. 10.1007/s00221-003-1743-7 14762637

[B229] Wegner D. M. Fuller V. A. Sparrow B. (2003). Clever hands: Uncontrolled intelligence in facilitated communication. *J. Pers. Soc. Psychol.* 85 5–19. 10.1037/0022-3514.85.1.5 12872881

[B230] Wehrenfennig A. Surian L. Wehrenfennig A. (2008). Autismo e comunicazione facilitata: Una rassegna degli studi sperimentali. *Psicol. Clin. Dello Sviluppa* 12 437–464. 10.1449/28487

[B231] Weierink L. Vermeulen R. J. Boyd R. N. (2013). Brain structure and executive functions in children with cerebral palsy: A systematic review. *Res. Dev. Disabil.* 34 1678–1688. 10.1016/j.ridd.2013.01.035 23500162

[B232] Weiss M. Moran M. Parker M. E. Foley J. (2013). Gait analysis of teenagers and young adults diagnosed with autism and severe verbal communication disorders. *Front. Integr. Neurosci.* 7:33. 10.3389/fnint.2013.00033 23730274 PMC3657630

[B233] Wertsch J. V. (1984). The zone of proximal development: Some conceptual issues. *N. Direct. Child Adolesc. Dev.* 1984 7–18. 10.1002/cd.23219842303

[B234] Wider C. Mitra S. Andrews M. Boulton H. (2020). Age-related differences in postural adjustments during limb movement and motor imagery in young and older adults. *Exp. Brain Res.* 238 771–787. 10.1007/s00221-020-05751-9 32107575 PMC7181438

[B235] Wider C. Mitra S. Boulton H. Andrews M. (2022). Age-related asymmetry in anticipatory postural movements during unilateral arm movement and imagery. *Exp. Brain Res*. 240, 2435–2457. 10.1007/s00221-022-06416-5 35930013 PMC9458590

[B236] Wollesen B. Voelcker-Rehage C. Regenbrecht T. Mattes K. (2016). Influence of a visual-verbal Stroop test on standing and walking performance of older adults. *Neuroscience* 318 166–177. 10.1016/j.neuroscience.2016.01.031 26808774

[B237] Wolpert D. M. Kawato M. (1998). Multiple paired forward and inverse models for motor control. *Neural Netw.* 11 1317–1329. 10.1016/S0893-6080(98)00066-5 12662752

[B238] Wolpert D. M. Miall R. C. Kawato M. (1998). Internal models in the cerebellum. *Trends Cogn. Sci.* 2 338–347. 10.1016/s1364-6613(98)01221-2 21227230

[B239] Woollacott M. Shumway-Cook A. (2002). Attention and the control of posture and gait: A review of an emerging area of research. *Gait Posture* 16 1–14. 10.1016/S0966-6362(01)00156-4 12127181

[B240] Wotherspoon J. Whittingham K. Sheffield J. Boyd R. N. (2023). Executive function, attention and autism symptomatology in school-aged children with cerebral palsy. *J. Dev. Phys. Disabil*. 10.1007/s10882-023-09905-9 [Epub ahead of print].

[B241] Wunsch K. Pfister R. Henning A. Aschersleben G. Weigelt M. (2016). No interrelation of motor planning and executive functions across young ages. *Front. Psychol.* 7:1031. 10.3389/fpsyg.2016.01031 27462285 PMC4940395

[B242] Yardley L. Gardner M. Bronstein A. Davies R. Buckwell D. Luxon L. (2001). Interference between postural control and mental task performance in patients with vestibular disorder and healthy controls. *J. Neurol. Neurosurg. Psychiatry* 71 48–52. 10.1136/jnnp.71.1.48 11413261 PMC1737444

[B243] Yeh T. T. Cluff T. Balasubramaniam R. (2014). Visual reliance for balance control in older adults persists when visual information is disrupted by artificial feedback delays. *PLoS One* 9:e91554. 10.1371/journal.pone.0091554 24614576 PMC3948884

[B244] Zanobini M. Scopesi A. (2001). La comunicazione facilitata in un bambino autistico. *Psicol. Clin. Svilupp.* 5 395–421. 10.1449/635

